# Restructuring of Cu-based Catalysts during CO Electroreduction: Evidence for the Dominant Role of Surface Defects on the C_2+_ Product Selectivity

**DOI:** 10.1021/acscatal.4c02718

**Published:** 2024-08-20

**Authors:** Floriane A. Rollier, Valery Muravev, Alexander Parastaev, Rim C. J. van de Poll, Jason M. J. J. Heinrichs, Bianca Ligt, Jérôme F. M. Simons, Marta Costa Figueiredo, Emiel J. M. Hensen

**Affiliations:** Laboratory of Inorganic Materials and Catalysis, Department of Chemical Engineering and Chemistry, Eindhoven University of Technology, P.O. Box 513, Eindhoven 5600 MB, The Netherlands

**Keywords:** CO electroreduction, Cu catalysts, C_2+_ products, CuO/Cu restructuring, surface structure, *in situ* WAXS, quasi-*in situ* XPS

## Abstract

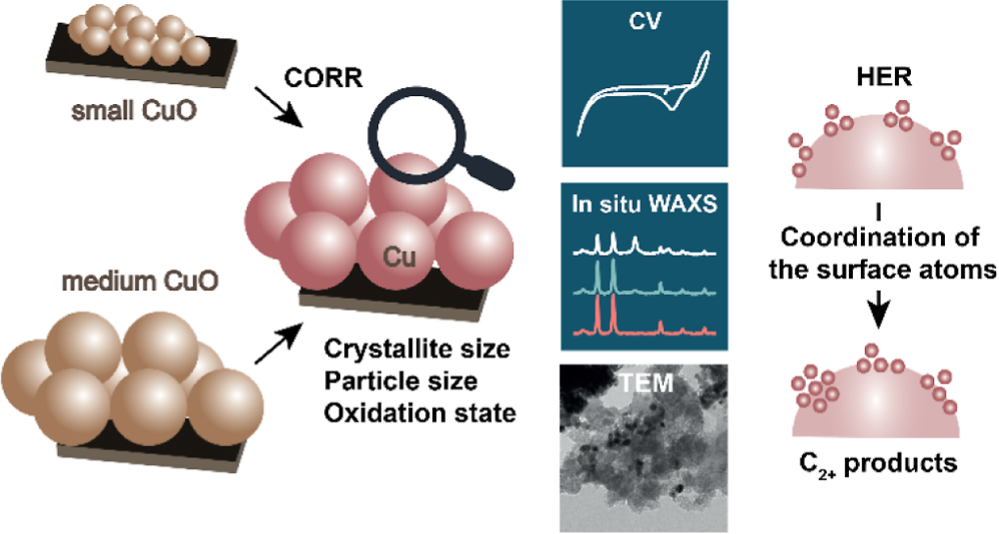

CO is the key reaction intermediate in the Cu-catalyzed electroreduction of CO_2_ to products containing C–C bonds. Herein, we investigate the impact of the particle size of CuO precursors on the direct electroreduction of CO (CORR) to C_2+_ products. Flame spray pyrolysis was used to prepare CuO particles with sizes between 4 and 30 nm. *In situ* synchrotron wide-angle X-ray scattering (WAXS), quasi-*in situ* X-ray photoelectron spectroscopy, and transmission electron microscopy demonstrated that, during CORR, the CuO precursors transformed into ∼30 nm metallic Cu particles with a crystalline domain size of ∼17 nm, independently of the initial size of the CuO precursors. Despite their similar morphology, the samples presented different Faradaic efficiencies (FEs) to C_2+_ products. The Cu particles derived from medium-sized (10–20 nm) CuO precursors were the most selective to C_2+_ products (FE 60%), while those derived from CuO precursors smaller than 10 nm displayed a high FE to H_2_. As the oxidation state, the particle and the crystallite sizes of these samples were similar after CORR, the differences in product distribution are attributed to the type and density of surface defects on the metallic Cu particles, as supported by studying electrochemical oxidation of the reduced Cu particles during CV cycling in combination with synchrotron WAXS. Cu particles derived from <10 nm CuO contained a higher density of more under-coordinated defects, resulting in a higher FE to H_2_ than Cu particles derived from 10 to 30 nm CuO. Bulk oxidation was most prominent and stable for Cu particles derived from medium-sized CuO, which indicated the more disordered nature of their surface compared to Cu particles derived from 30 nm CuO precursors and their lower reactivity compared to Cu particles derived from small CuO. Cu particles derived from <10 nm CuO initially displayed intense redox behavior, quickly fading during subsequent CVs. Our results evidence the significant restructuring during the electrochemical reduction of CuO precursors into Cu particles of similar size. The differences in CORR performance of these Cu particles of similar size can be correlated to different surface structures, qualitatively resolved by studying surface and bulk oxidation, which affect the competition between CO dimerization to yield C_2+_ products and undesired H_2_ evolution.

## Introduction

The release of large amounts of CO_2_ into the atmosphere due to the widespread use of fossil fuels has led to substantial concerns about climate change.^1−3^ To avoid an environmental crisis, it is necessary to develop strategies that decrease the use of nonrenewable fossil resources to produce fuels and chemicals. Closing the carbon cycle by transforming waste CO_2_ into value-added compounds using renewable energy is a potential long-term solution.^4,5^ The electrochemical reduction of CO_2_ (CO2RR), studied since the early 1990s, can selectively yield two-electron transfer products, such as formic acid (HCOOH) and carbon monoxide (CO).^4,6,7^ While HCOOH is a potential liquid energy carrier, CO is a versatile feedstock for producing various chemicals, including those with C–C bonds (C_2+_ products). The direct CO2RR to C_2+_ products has also gained attention but suffers from poor selectivity due to complex mechanisms requiring multiple proton-coupled electron transfer steps. Mechanistically, it is thought that CO_2_ first undergoes a two-electron transfer, forming a *CO intermediate, which can be further reduced to C_2+_ products such as ethylene, ethanol, and propanol.^8−10^ These products are important chemical building blocks and dense energy-carriers.^4,11^ As direct CO2RR chemistry to C_2+_ remains very challenging, a sequential process involving CO_2_ reduction to CO followed by CO electroreduction to C_2+_ products might be beneficial.^12−14^ Haldor Topsøe commercialized solid-oxide electrochemical CO2RR technology to produce CO.^15−17^ The high-purity CO product can be used in subsequent hydrogenation and/or reduction reactions to obtain valuable products with C–C bonds.

The direct electrochemical reduction of CO (CORR) has been less studied than CO2RR. Despite this, the selectivity to C_2+_ products is typically higher for CORR than CO2RR.^10,12^ It has been proposed that a high CO surface coverage and the smaller number of electrons to be transferred benefit C–C bond formation.^18,19^ Cu presents a unique performance in the formation of C_2+_ products in CORR and CO2RR.^20−24^ The optimal binding strength of *CO and *H to Cu is thought to facilitate C–C coupling reactions and product desorption, whereas other metals having weak *CO binding strength (*e.g.*, Ag, Au, Zn) or strong *H binding strength (*e.g.*, Co, Ru, Pt) do not form C_2+_ products.^21,23,25,26^ As the CO_2_ to CO step can be carried out with high selectivity,^15,27,28^ it is important to further optimize CORR to C_2+_ products for practical applications, with insights for CORR being also relevant for CO2RR.^10,13^

Despite the unique electrocatalytic properties of Cu, one of the main challenges in CO2RR and CORR remains its poor selectivity toward specific products with C–C bonds.^26^ The competing hydrogen evolution reaction (HER) presents another challenge, negatively affecting the Faradaic efficiency (FE) to C_2+_ products. Both aspects have been recognized as key hurdles toward practical implementation of this technology.^6^ Recent studies highlighted the critical role of Cu surface topology on the selectivity of CO2RR.^20,29−32^ Especially the density of under-coordinated surface Cu atoms affects the formation of C_2+_ product and H_2_ during CO2RR and HER.^33−35^ While under-coordinated Cu surface sites increase the formation of C_2+_ products compared to terraces, a coordination number that is too low for the surface Cu atoms promotes the competing HER. For instance, the groups of Roldan Cuenya and Strasser demonstrated that Cu particles smaller than 15 nm present a higher selectivity toward H_2_, contrasting with the higher selectivity to C_2+_ products of bulk Cu (*e.g.*, polycrystalline metal foil) in CO2RR.^35^ This difference was attributed to variation in the density of low- and high-coordinated surface atoms, the former prevailing in small nanoparticles.^35^ On the contrary, the group of Yang used operando electrochemical STEM (EC-STEM) and *ex situ* XAS to demonstrate that under-coordinated Cu sites at grain boundaries were responsible for the formation of C_2+_ products.^34^ These studies illustrate that the optimum Cu surface topology for C_2+_ product formation may involve Cu surface atoms with intermediate reactivity. Resolving the surface structure on metallic electrocatalysts during the reaction remains very challenging. The combination of complementary *ex situ* and *in situ* characterization tools (*e.g.*, microscopy, grazing incidence XRD, XAS) with electrochemical methods can be used to probe differences in surface reactivity.^36,37^

Compared to CO2RR, the impact of morphology and particularly of particle size on CORR has been much less investigated and remains essentially underexplored.^33,38,39^ Given the pivotal role of CO in forming C_2+_ products and the relevance of the two-step process presented earlier, the study of such an effect is pivotal. The poor solubility of CO in aqueous electrolytes makes the use of bulk electrodes, such as foils, problematic.^40,41^ Therefore, the use of powder catalysts immobilized on gas diffusion electrodes (GDEs) is required to maintain a constant supply of the CO reactant to the liquid–solid interface, thereby avoiding mass transport limitations.^42^

In the present study, we employed flame spray pyrolysis (FSP) to synthesize CuO nanoparticles of different sizes. The size of the as-prepared CuO precursors deposited on GDEs strongly impacted the catalytic performance in CORR, with very small particles (4 nm) promoting HER and medium particles (10 nm) being more selective to C_2+_ products. *In situ* wide-angle X-ray scattering (WAXS) and quasi-*in situ* X-ray photoelectron spectroscopy (XPS) studies highlight the extensive structural changes of the CuO precursors during their reduction to Cu under CORR conditions. Specifically, the CuO precursors yielded to Cu metal particles of comparable size, independently of their initial sizes. Electrochemical oxidation of the surface of the reduced Cu particles in combination with WAXS was used to determine the different surface reactivities of these Cu particles derived from differently sized CuO precursors. Requirements for optimum C_2+_ product formation will be discussed.

## Methods and Materials

### Catalyst Synthesis

Flame-spray pyrolysis (FSP) was used to synthesize CuO nanoparticles (Figure S1). The preparation of the solutions was derived from existing literature.^43^ A commercial Tethis NPS10 setup was used for FSP synthesis. Cu(NO_3_)_2_·3H_2_O was dissolved in 1 to 1 (vol.) mixture of absolute ethanol and 2-ethylhexanoic acid. The concentration of the solution was 0.13 M. The mixture was stirred and heated up to 80 °C until the full dissolution of the salt. The mixture was then injected through the nozzle of the FSP system into a methane-oxygen flame (1.5 L/min CH_4_ and 3 L/min O_2_). The pressure drop at the nozzle was set to 2.5 bar. Particles were collected from a quartz filter using a spatula. The catalysts were afterward sieved (450 μm) to remove the quartz fibers. The injection flow and dispersion flow rates were varied to control the size of the synthesized CuO nanoparticles (Table S1).

### Electrode Preparation

The catalyst was deposited on a gas diffusion layer (GDL) (SIGRACET 22BB) to prepare an electrode using a drop-casting technique. An ink containing 10 mg of catalyst, 120 μL of Nafion solution (5 wt %), and 800 μL of absolute ethanol was prepared and sonicated for 20 min to disperse the nanoparticles. The dispersion was then drop-casted onto the GDL and dried naturally in air overnight. The catalyst loading was 2 mg/cm^2^. The above ink proportions are calculated for a 5 cm^2^ electrode.

### Characterization

*WAXS* was used to analyze the crystalline structure of the samples with better precision than lab-based XRD. The measurements were carried out at the beamline ID 31 of the ESRF synchrotron radiation facility. An X-ray energy of 75 keV (λ = 0.0165 nm) and a Pilatus CdTe 2 M detector were used. The powder samples were measured in Kapton capillaries. The *in situ* WAXS measurements and the *in situ* cell (Figure S35) are described in detail in the Supporting Information file.

*XPS* was utilized to determine the surface chemical state and the surface composition of the fresh and used samples. The *ex situ* experiments were performed on a Thermo Scientific K-alpha spectrometer using an Al-Kα X-ray source (1486.6 eV, 72 W). The quasi *in situ* experiments were performed on a SPECS system using the same X-ray energy (1486.6 eV, 50 W) and these measurements are described in detail in the Supporting Information file. Calibration on the C–C component in the C 1s spectra (284.5 eV) was applied during data treatment. Cu 2p_3/2_ and Cu LMM were fitted using the models reported by Biesinger.^44^

Transmission electron microscopy (*TEM*) was carried out on an FEI Technai microscope (Sphera) (as-prepared samples, used 6, 10, and 30 nm samples) and a Glacios CryoTEM (used 4 nm sample). The acceleration voltage was 200 kV. The fresh catalyst powders were dispersed in ethanol before deposition on a TEM grid. The used catalysts were recovered from the GDE. After the reaction, the electrode was rinsed with ultrapure water and a few drops of absolute ethanol were placed on its surface. The catalyst was removed from the carbon paper using a spatula. The particles dispersed in ethanol were then drop casted on TEM Cu-grids. Data treatment was performed with the ImageJ software.

### Catalytic Performance—CORR

The catalytic performance for CORR was measured in a leak-tight H-cell. The catalyst deposited on a carbon paper forms the GDE/working electrode. Pt foil and RHE were used as counter and reference electrodes, respectively. The RHE was placed 2 mm away from the working electrode surface. Working and counter electrodes had a geometrical surface area of 1 cm^2^ each. A Metrohm AUTOLAB PGSTAT302N potentiostat was used in the experiments. The catholyte was continuously flushed with 15 mL/min of CO in a flow-through configuration. The CO and gaseous products were directed toward a mass-spectrometer [Pfeiffer vacuum (Balzers instruments) Thermostar GSD 300 T2] and a gas chromatography apparatus (GC TRACE 1300—Thermo Fischer Scientific) for online analysis. The flow out of the cell and the pressure were monitored by a mass flow meter (Bronkhorst) and a manometer, respectively. The 23 mL of electrolyte placed in each compartment were static. Liquid products were analyzed by ^1^H NMR after the reaction. Additional details on product quantification and FE calculations are described in the Supporting Information file.

## Results and Discussion

### Characterization of As-synthesized Catalysts

**Figure 1:**
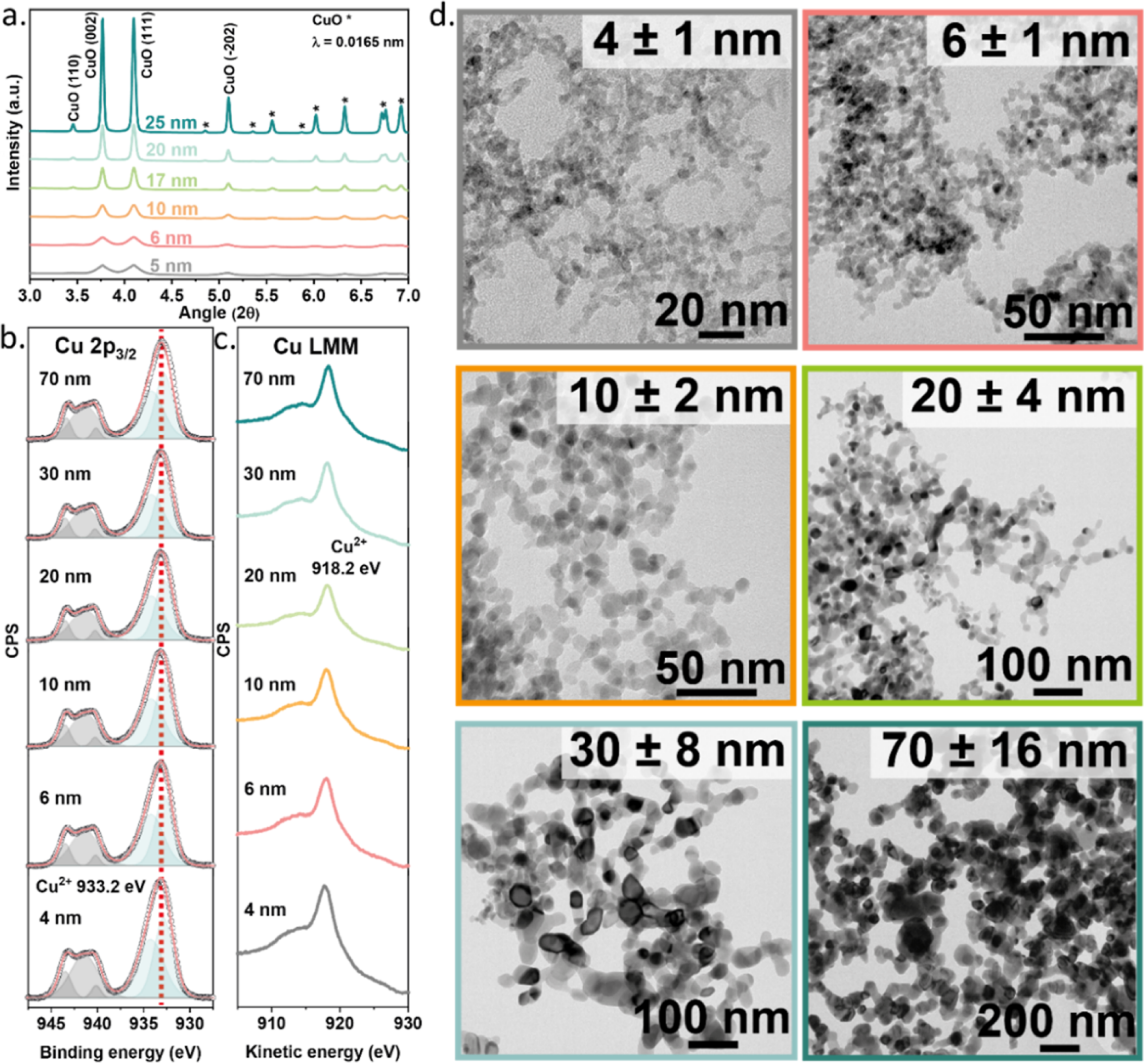
Characterization of the as-synthesized CuO samples. (a) WAXS of the as-synthesized catalysts (λ = 0.0165 nm), (b,c) XPS spectra of Cu 2p_3/2_ (b) and Cu LMM (c) regions, (d) TEM pictures of the different particle sizes.

Size-controlled CuO nanoparticles were synthesized by FSP (Figure S1). The FSP synthesis parameters were tuned to obtain a range of CuO particle sizes (Table S1). Synchrotron-based WAXS was employed to characterize the crystalline phases and the crystalline domain sizes of the as-synthesized samples (Figure 1a). The diffraction patterns show that the samples were composed of monoclinic CuO (Figure 1a).^45^ Crystallite sizes were determined by applying the Scherrer equation to the CuO (111) and (002) reflections. Variation of the FSP synthesis parameters led to CuO samples with crystallite sizes between 5 and 20 nm (Table S1). Decreasing the flow at which the precursor solution was injected in the flame or increasing the dispersion flow around the flame resulted in smaller CuO crystallites.^46^ The particle sizes of the as-prepared CuO samples were determined by TEM (Figures 1d and S3). The TEM particle sizes of the 4 to 20 nm CuO samples agreed well with the crystallite sizes measured by WAXS (5 to 17 nm), indicating that the particles were monocrystalline (Table S1). The small and medium particles exhibited a narrow size distribution (Figure S3). Conversely, the TEM particle size of the largest FSP-prepared CuO sample, 30 nm, deviated substantially from the crystallite size determined by WAXS (20 nm). We stress that the size of a particle is not necessarily the same as that of a crystallite, as particles can be composed of several crystallites. Moreover, it cannot be excluded that part of a particle is amorphous. It is likely that the high injection rate during FSP synthesis of the 30 nm sample, forming larger precursor droplets, caused the solid particles to sinter and form relatively large polycrystalline 30 nm CuO particles.^46,47^

To prepare a CuO sample with a substantially larger particle size of 70 nm (Figure 1d), a portion of the 10 nm CuO sample was calcined at 450 °C for 3 h. The as-prepared samples were further characterized by XPS (Figure 1b,c).

The Cu 2p_3/2_ line at a binding energy of 933.2 eV and the distinct satellite features in the 940–945 eV range showed the exclusive presence of Cu^2+^ at the surface of these samples. In line with this, the maximum of the Cu LMM Auger spectra was at ∼918.2 eV, characteristic of Cu^2+^ (Figure 1c).^44^ Thus, WAXS and XPS demonstrated the formation of CuO particles.

### CO Electroreduction

**Figure 2:**
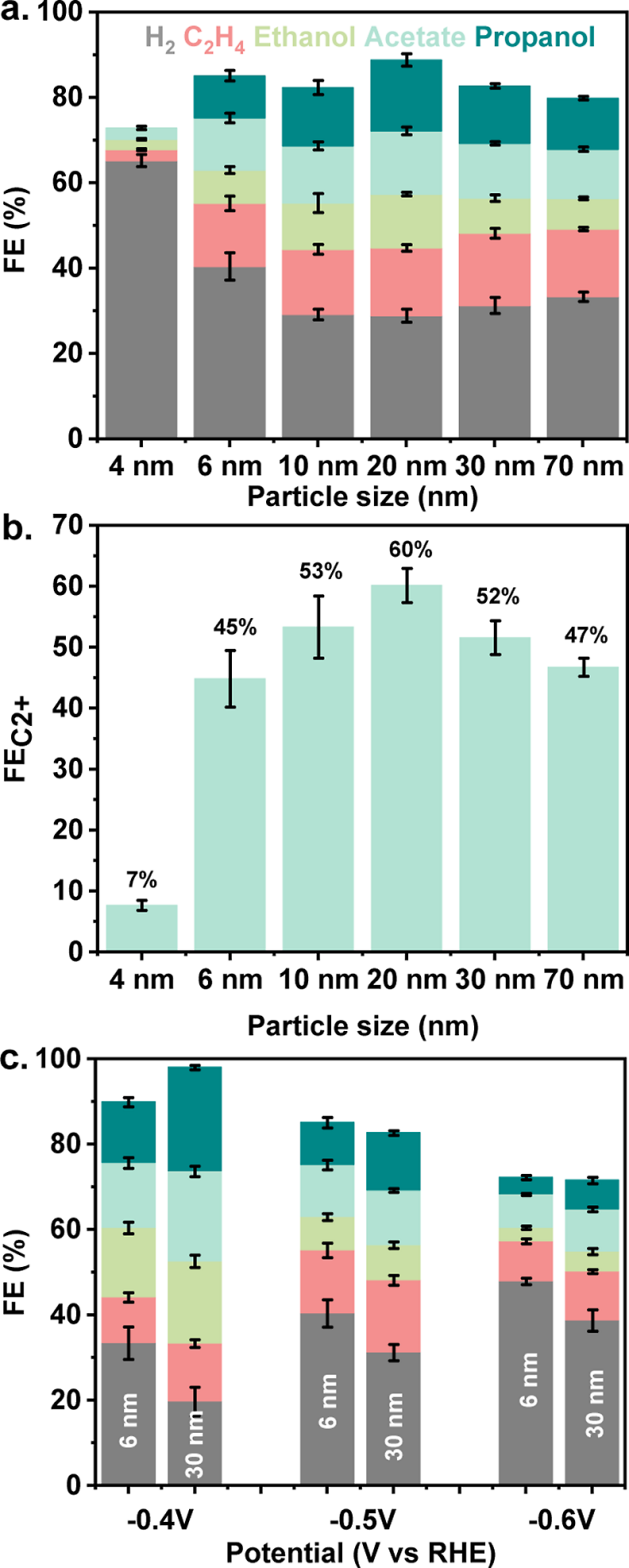
CORR performance of CuO precursors of different size. (a) FE recorded at −0.5 V *vs* RHE for all particle sizes, (b) FE C_2+_ recorded at −0.5 V *vs* RHE and (c) FE recorded on small (6 nm) and large (30 nm) particles at potentials ranging from −0.4 to −0.6 V *vs* RHE.

The CORR performance of the as-synthesized samples was evaluated at a potential of −0.5 V *vs* RHE (reversible hydrogen electrode) for 1 h in 3 M KOH electrolyte. The experiments were conducted in an H-cell equipped with a GDE (GDE-modified H-cell) to ensure a constant supply of CO to the catalyst surface. FEs were significantly impacted by the initial size of the CuO precursors (Figures 2a and S8). The smallest CuO sample with a size of 4 nm favored the formation of H_2_ with a FE of 65%. The group of Strasser related the high H_2_ selectivity of small Cu particles to a large number of low-coordinated surface atoms (CN < 8, CN = coordination number).^35^ The H_2_ FE for the 6 and 10 nm CuO samples decreased to 40 and 29%, respectively, and leveled off at 31 ± 2% for even larger particles. In line with this, the H_2_ FE of the calcined sample containing 70 nm CuO particles was 33%. The FE to C_2+_ products also depended on the initial CuO particle size (Figures 2a,c and S8). The C_2+_ product FE followed an opposite trend compared to the H_2_ FE due to the competition between CORR and HER on Cu-based catalysts.^4,18,43−45^ The C_2+_ product FE was only 7% for the 4 nm CuO sample, gradually increasing with the CuO particle size. While 6 nm CuO sample exhibited a C_2+_ product FE of 45%, the 20 nm CuO sample displayed a high FE of 60%. The lower density of low-coordinated surface atoms likely suppressed H_2_ formation, which can explain the formation of more C_2+_ products.^35^ Based on the observed CORR performance, we speculate that the Cu surface on medium and large particles benefits the formation of C_2+_ products at the expense of HER, which may be correlated to a lower abundance of under-coordinated sites on these particles.^35,48,49^ Besides the coordination of the surface atoms, the surface structure and oxidation state may also impact the product distribution on Cu-based catalysts.^33,39,50,51^ These aspects will be discussed below. The C_2+_ products formed in CORR were hydrocarbons like ethylene and oxygenates like acetate, ethanol, and propanol. As shown in Figures 2a and S8, only small variations of the ethylene FE were observed for the CuO samples with particle sizes between 6 and 70 nm. Conversely, increasing the size of CuO samples from 20 to 30–70 nm or decreasing the size of CuO samples from 20 to 6 nm negatively impacted the selectivity toward C_2+_ oxygenates. Assuming that CO2RR and CORR share similar reaction pathways for C–C coupling from CO,^52−55^ we can expect that the high pH of the electrolyte used in our study favors *CO–*CO dimerization over other possible C–C coupling mechanisms, regardless of the particle size. It is, for instance, known that alternative pathways to C_2+_ products, such as *CO–COH coupling, a carbene-like mechanism involving *CH_2_, and CO insertion, are inhibited under alkaline conditions.^8,52,55,56^ The formation of oxygenates and hydrocarbons on small, medium, and large CuO particles likely originated from the same *CO–*CO dimer, despite the different products FEs observed. Ethylene is formed from the *CO–*CO dimer through a different mechanistic pathway than acetate.^4,57^ Acetate-like intermediates are precursors for the formation of ethanol and propanol.^57,58^ In this work, the CuO samples with particle sizes from 6 to 70 nm exhibit similar selectivity to ethylene, whereas the selectivity to acetate, ethanol, and propanol varied strongly with particle size. As the chemical and physical properties of the Cu-based catalysts (*e.g.*, particle size, presence of defective sites, oxidation state, *etc.*) are known to influence the product distribution,^29,59^ we speculate that the particles may present different surface properties under CORR conditions. These differences may not affect the carbophilicity of the surface, which is necessary for ethylene formation. At the same time, they impact the oxophilicity of the surface, which can contribute to stabilizing the intermediates involved in the formation of oxygenates.^57^ Finally, it is known that the high alkalinity of the electrolyte blocks C_1_ formation pathways, explaining why only small amounts of C_1_ products, such as methane, were obtained in this study (Figures S9, S11 and Note S2).^53^ The current densities, normalized by electrochemically active surface area (ECSA) (Figures S5 and S6), were also found to depend on the particle size (Figure S10a). Similar to the FE trends, the H_2_ partial current density was high on small particles (4 nm; −0.08 mA/cm^2^) and low on medium and large particles (20 nm; −0.02 mA/cm^2^). Conversely, the C_2+_ product current densities were the highest on medium and large particles (*e.g.*, 20 nm; −0.04 mA/cm^2^). We evaluated the influence of the applied potential from −0.4 to −0.6 V *vs* RHE on the CORR performance of the 6 nm (referred to as small particles) and 30 nm particles (referred to as large particles) (Figures 2c and S10b,c). At low potential (−0.4 V *vs* RHE), the FE to C_2+_ products reached 57 and 78% for small and large particles, respectively. The FE for propanol and acetate of the large particles were 24 and 21%, respectively, while a relatively low H_2_ FE of 19% was measured. Conversely, an H_2_ FE of 34% was recorded on small particles. Previous research demonstrated that C–C coupling reactions are potential-dependent.^9,60,61^ As mentioned above, alkaline conditions favor *CO–*CO dimerization followed by proton-coupled electron transfer steps over other C–C coupling pathways.^8,52,55,56^ Moreover, a high pH of the electrolyte suppresses H_2_ evolution, which benefits the C_2+_ selectivity, especially at low potential. At intermediate potential (−0.5 V *vs* RHE), the H_2_ selectivity increased to 40 and 31% on small and large particles, respectively, at the expense of the C_2+_ oxygenates FE. HER and CORR current densities were higher at this potential than at −0.4 V *vs* RHE (Figure S10b,c), indicating faster H_2_ and C_2+_ formation. Competition between *CO and *H on the active sites determines the selectivity.^62,63^ As a result of this competition, we expect an increase in the H coverage and a decrease in the CO coverage with decreasing potential, explaining the decrease in the C_2+_ product FE.^62,63^ The ethylene FE was, however, higher at −0.5 V *vs* RHE compared to the FE at less negative potential. A higher H coverage can facilitate the removal of oxygen-containing groups in reaction intermediates, resulting in products like ethylene. At a more negative potential of −0.6 V *vs* RHE, the selectivity toward C–C containing oxygenates and hydrocarbons dropped sharply on large particles, favoring H_2_ formation with a FE of 39%. Under these conditions, we can expect H and CO adsorption to compete, which can explain the increase of the H_2_ FE at the expense of the FE to C_2+_ products. Similar trends were observed for the 6 nm CuO catalyst. Nevertheless, the changes in the FEs with a potential decrease from −0.4 to −0.5 V *vs* RHE were less pronounced for the small particles. The overall higher selectivity toward H_2_ on small particles is the likely reason for the lower impact of the potential on the product distribution. A comparison of the current densities as a function of particle sizes showed that the total current densities measured on small and large CuO samples were similar at all potentials. Yet, small CuO particles were substantially less active for C_2+_ production than large ones (Figure S10b,c). An opposite trend was observed for H_2_ production.

The CORR performance stability of the 6 and 30 nm samples was tested over 5 h (Figure S12 and Note S3). While the C_2+_ products FE decreased over time for both samples, the 30 nm sample was most impacted, showing a decline from 47% in the first hour to 29% after 5 h. The CORR performance of both samples was similar after 5 h. An increase in the ECSA was recorded during this time (Figures S12 and S13), pointing to the continuous restructuring of the catalysts during CORR. In the next section, we investigate the restructuring of the catalysts during the CV pretreatment and subsequent CA measurements.

### Structural Transformation of CuO Nanoparticles under Reducing Conditions

**Figure 3:**
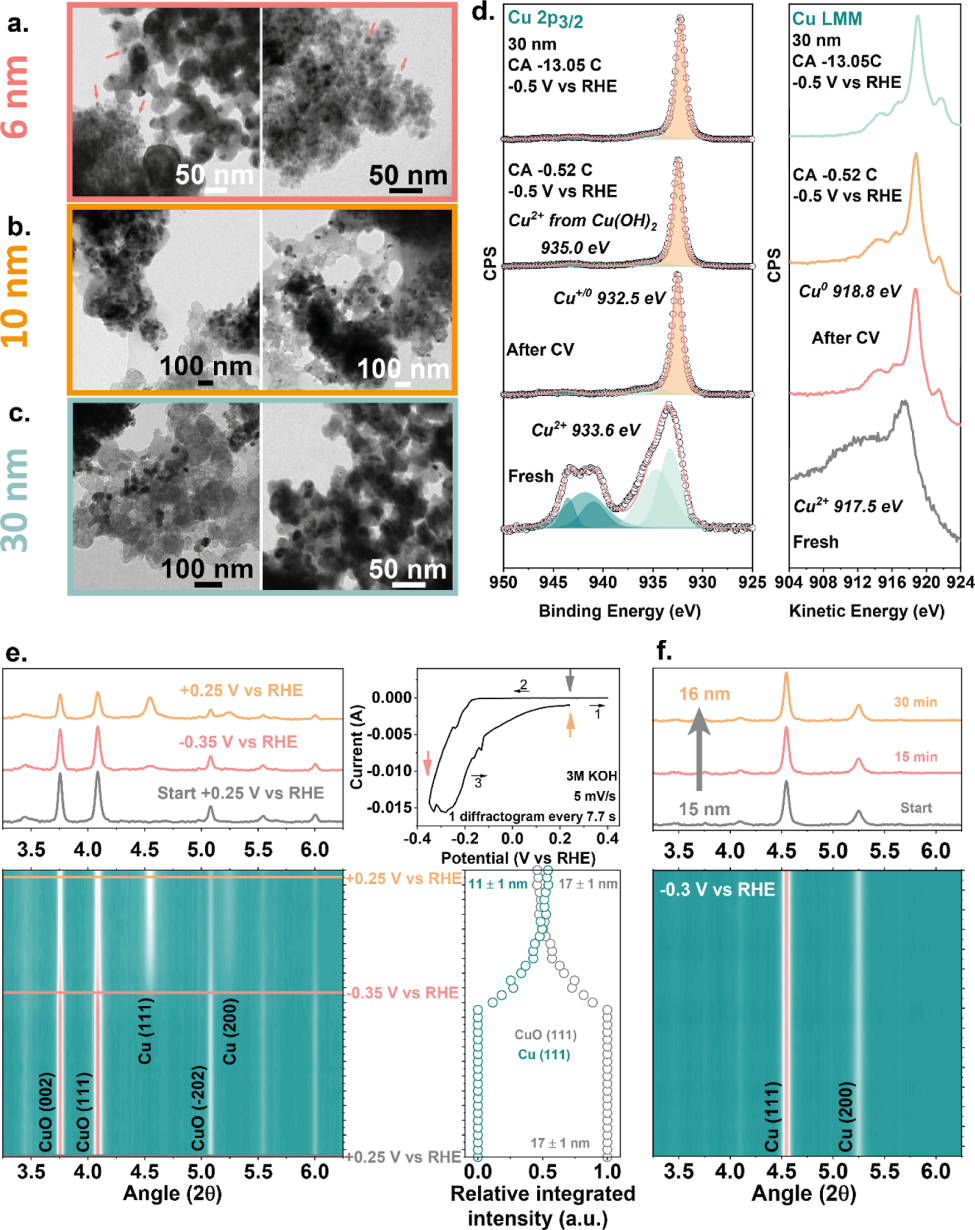
Characterization of electroreduced catalysts. (a) TEM images of used 6 nm CuO particles (the pink arrows point to the 9 nm particles), (b) TEM images of used 10 nm CuO particles, (c) TEM images of used 30 nm CuO particles, (d) quasi-*in situ* XPS spectra: Cu 2p_3/2_ and Cu LMM lines of 30 nm CuO particles, (e) *in situ* WAXS during the first CV of 30 nm CuO particles and (f) *in situ* WAXS during the chronoamperometry (CA) of 30 nm CuO particles.

The CuO nanoparticles are expected to reduce under CORR conditions.^64^ As a result, the chemical and physical properties of the precursors change during the reaction, and correlations between *ex situ* probed structures and the electrocatalytic performance are less relevant. Therefore, investigating the state of the catalyst under CORR conditions is essential to correlate the activity to C_2+_ products to the structure.^65,66^ For this purpose, we investigated the samples during and after CORR. TEM images of used samples containing initially 4, 6, 10, and 30 nm CuO particles after 1 h CORR are shown in Figures 3a–c and S14. After CORR, the catalyst was recovered by lightly scratching the surface of the electrode with a spatula in ethanol. This led to the removal of Nafion and carbon particles from the GDL. The sensitivity of Nafion to the electron beam made TEM imaging challenging. The carbon particles and the Nafion can nevertheless be distinguished in the TEM images as irregular and gel-like structures, respectively, which contrast with the spherical Cu particles. During CORR, the initially very small sample (CuO 4 nm) underwent substantial sintering, resulting in particles with an average size of 27 nm (Figures S14 and S15). The particle size distribution was broad, with most of the particles being between 10 and 40 nm, although smaller and larger particles were also observed. The initial 6 nm CuO sample evolved into particles with a bimodal size distribution during CORR. One population had only a slightly larger particle size of 9 nm (pink arrows in Figure 3a) compared to the initial size of 6 nm, while the other population grew to sizes between 20 and 50 nm (Figure S15). The average particle size of the sample was 20 ± 14 nm. Sintering of metal particles, resulting in the reduction of their surface tension, is a common phenomenon.^67−69^ The observation of a bimodal size distribution can be because not all the catalyst in the GDE was involved to a similar extent in CORR. The dissolution/redeposition of Cu at the surface of the catalyst layers, especially pronounced in CO-rich environments,^70^ can explain the growth of the nanoparticles at the surface. Under CO2RR conditions, particle growth is reported to follow an Ostwald ripening-like mechanism.^70^ We speculate that the large particles (20–50 nm) were located at the very surface of the electrode, and their growth was enhanced through the redeposition of dissolved Cu cations located in the vicinity of the electrode. The smaller particles (9 nm) possibly resided in confined areas within the electrode and were less accessible for the redeposition of dissolved Cu cations. The amount of Cu dissolved in the electrolyte after CORR, determined by ICP-OES elemental analysis, was negligible for all samples (Table S3), indicating the minor loss of materials during the reaction, likely due to the fast redeposition of Cu cations. The initially 10 nm CuO sample also evolved into larger particles but in a more homogeneous manner with an average size of 30 ± 12 nm (Figure S15). During 1 h CORR, the initially 30 nm CuO sample evolved into 30 ± 8 nm nanoparticles of metallic Cu nature, as will be shown later. This demonstrates that restructuring affected all samples during the reduction of CuO precursors. Our data indicate that the metallic Cu particles reach a size of ca. 30 nm under the applied electrochemical conditions, irrespective of the initial CuO particle size. Further, the growth seems suppressed once the particles have reached such an average size.

To further study the impact of the reducing conditions on the particle size, we employed *in situ* WAXS. CuO samples with 4 nm (very small), 6 nm (small), 10 nm (medium), and 30 nm (large) particles were studied by cyclic voltammetry to mimic the pretreatment performed before the CORR measurements. During the first CV cycle (start/stop at +0.25 V *vs* RHE; CV from +0.4 V *vs* RHE to −0.35 V *vs* RHE, 5 mV/s), all catalysts underwent partial reduction of CuO to metallic Cu (Figures 3e, S16, S19a, and S21a). When a reducing potential of −0.3 V *vs* RHE was reached during the CV of the large sample, the (002) and (111) reflections of the CuO phase faded away (Figure 3e). Simultaneously, characteristic metallic Cu (111) and (200) reflections appeared in the diffractograms.^71^ The intensity of these Cu metal reflections increased throughout the CV cycle. The phase evolution was followed by comparing the integrated intensities (areas) of the (111) reflections of CuO and Cu. The relative integrated intensity of Cu (111) is given as the integrated intensity of this reflection divided by the sum of the integrated intensities of CuO (111) and Cu (111). We clarify that the relative intensities do not reflect the amounts of the phases but provide an estimate of the degree of reduction. At the start of the first CV, only CuO was present (Figure 3e). The reduction of CuO to Cu, observed from −0.3 V *vs* RHE in the diffractograms, resulted in a sharp increase of the relative Cu (111) integrated intensity. At the end of the first CV, the relative Cu (111) integrated intensity reached 0.54 for the large sample, indicating that only part of CuO was reduced (Figure 3e).

The potential at which metallic Cu became visible in the diffractograms differed for the other samples. For instance, reflections of the metallic Cu phase were visible from −0.25 V *vs* RHE (cathodic sweep) in the diffractograms of the 10 nm sample. The current recorded at this potential was −8 mA (Figure S21a). For the 6 and 4 nm samples, metallic Cu appeared from −0.17 V *vs* RHE (anodic sweep) and −0.33 V *vs* RHE (anodic sweep), respectively. These were associated with lower currents than for the 10 and 30 nm samples (−8 mA for 30 nm, −3 mA for 6 nm, and −7 mA for 4 nm) (Figures 3e, S16, S19a, and S21a). The differences in the total current likely caused the change in the potential where metallic Cu was formed, indicating that a certain amount of charge is used before these crystallites are detected by WAXS (Figure S24). Recent works suggest that the redox properties of transition metal oxides, such as CoO_*x*_, may depend on the particle size.^72^ To the best of our knowledge, the size-dependent electrochemical reducibility of CuO has not yet been studied. In thermal catalysis, CO temperature-programmed reduction showed that small CuO particles reduced at lower temperatures than large particles.^73^ As the reduction currents cannot be compared in an absolute sense, our data cannot provide an unequivocal insight into the impact of particle size on the reduction of CuO.

Under the reducing conditions of the first CV, a typical polycrystalline Cu phase was formed in all the samples without indications of preferential faceting.^71^ The Cu metal crystallite sizes were determined by applying the Scherrer equation to the Cu (111) reflections. The widths and positions were obtained by fitting the diffraction peaks using a Voigt function, which accounts for instrumental and material-related broadening.^74,75^ At the end of the first CV, the crystallite size of metallic Cu was comparable for all samples (11 to 13 nm). After the first cycle, all catalysts comprised a mixture of CuO and Cu. No evidence of Cu_2_O was observed in the diffractograms, implying that CuO directly transformed into Cu or that any Cu_2_O formed was either amorphous or in low concentration, and thus invisible by WAXS.^76^ During subsequent cycles, the reduction of CuO to Cu proceeded further. The Cu (111) relative integrated intensity increased from 0.54 to 0.9 for the 30 nm particles between the end of the first and last CV cycles (Figures 3e and S23). In the last CV cycle, the amount of metallic Cu did not change for the 4, 10, and 30 nm samples, as the relative integrated intensity of Cu (111) was nearly constant at values between 0.9 and 1. On the contrary, the reduction was still progressing for the 6 nm sample, as follows from the small amount of CuO observed at the start of the last cycle. Overall, after 6 CV cycles, only traces of CuO remained with the metallic Cu (111) and (200) reflections dominating the diffraction patterns of all the samples (Figures S17, S19b, S21b, and S23). Therefore, the pretreatment by CV cycling led to similar CuO reduction degrees, independent of the initial CuO particle size.

The chronoamperometry (CA) measurements performed after these CV cycles (Figures 3f, S18, S20, S22, and S25) show that the catalysts remained reduced under a constant negative potential of −0.3 V *vs* RHE. During these measurements, the crystallite size of Cu, estimated using the Cu (111) reflections, slightly increased to 16–17 nm for all the catalysts (Figures 3f, S18, S20, S22, and S25). The resulting Cu crystallite sizes were similar for all samples and independent of the initial CuO size. We, therefore, conclude that neither the initial size of the CuO particles nor the size of the *in situ*-formed Cu crystallites can explain the differences in C_2+_ product FE measured during CORR. Instead, different surface topologies (surface facets, defects) or oxidation states may explain the observed differences in the catalytic performance.^77^

Previous research linked the density of grain boundaries in as-prepared Cu-based catalysts to higher C_2+_ product FE.^33,60^ The comparison of particle sizes determined by TEM with the crystallite sizes of as-prepared catalysts estimated by WAXS indicated that our large CuO particles contained more grain boundaries than small particles. Yet, postreaction TEM and *in situ* WAXS revealed that all samples have a comparable particle and crystallite size after reduction, suggesting a similar density of grain boundaries. This parameter can, therefore, not explain the enhanced C_2+_ product FE observed on medium and large samples in CORR compared to the small sample. As the surface oxidation state and structure were also reported to influence the formation of C_2+_ products on Cu-based catalysts, we investigated whether these surface properties caused the different CORR performances displayed by the samples, comprising metallic Cu particles with similar sizes after reduction. These differences in surface structures and/or compositions may be derived from how the CuO precursors restructure into Cu particles under reduction, which likely depends on the initial CuO size.

The surface oxidation state was investigated using surface-sensitive quasi-*in situ* XPS analysis after electroreduction. By carrying out the electrochemical measurements in a cell connected to the XPS, the used sample was transferred into the XPS instrument under an inert He atmosphere, avoiding air exposure. This prevented reoxidation, typically occurring in Cu-based catalysts during conventional *ex situ* approaches. After purging the electrolyte for 20 min with He, 4 CV cycles were carried out from −1.0 to +0.5 V *vs* RHE to reproduce the pretreatment set before the CORR performance measurements. The Cu 2p_3/2_ spectra of the 30 nm CuO sample before and after CV cycling are shown in Figure 3d. The initially dominant Cu^2+^ component from CuO, located at a binding energy of 933.6 eV, was absent after CV cycling. A new contribution, corresponding to Cu^+^ or Cu^0^, emerged at 932.5 eV.^44^ The Cu LMM Auger region was also measured to discriminate one from another. The maximum of the Cu LMM region is at 918.8 eV, and the line shape points to the predominance of metallic Cu. The absence of an additional feature at 916.8 eV confirmed that there were few or no Cu^+^ species after CV cycling. A comparison of the Auger regions for small and large particles showed that the line shape around 916.8 eV was similar for both samples, indicating no significant differences in the possible Cu^+^ content. On the contrary, a small fraction of Cu^2+^ remained, as evidenced by fitting the Cu 2p_3/2_ spectra (Figures 3d and S26). This small contribution of Cu(OH)_2_ located at ∼935 eV (11–18% of Cu 2p_3/2_) likely originated from the reaction of reduced Cu surface with the OH^–^ present in the electrolyte during drying in vacuum or at open circuit potential (OCP).^78,79^ The resulting Cu^2+^ species from Cu(OH)_2_ were also visible in the Cu LMM Auger region at 914.5 eV. Thus, quasi-*in situ* XPS confirmed the predominantly reduced nature of the surface and pointed out the similar oxidation state of the samples after CV cycling.

To further investigate the oxidation state of the surface during CA, a constant potential of −0.5 V *vs* RHE was applied until charges of −0.5C and −13.0C were exchanged (Figures 3d and S26). The XPS data show that the 6 and 30 nm CuO samples exhibited the same high reduction degree, regardless of the duration of the CA measurement. The Cu 2p_3/2_ and Cu LMM spectra revealed the reduction of CuO to Cu metal, with small amounts of Cu(OH)_2_ (∼15%) likely formed at the OCP. Moreover, the Cu LMM line shapes of small and large samples, suggesting the absence of Cu^+^, were similar after CA. As the surface and the bulk of all samples were reduced to a similar extent independently of the initial CuO particle size, we conclude that the oxidation state of Cu cannot explain the higher C_2+_ product FE obtained with the large particles and will further investigate the differences in surface structure.

### Electrochemical Oxidation of the Reduced Cu Particles

It is challenging to characterize the surface topology of Cu-based nanoparticles during CO2RR and CORR. A technique like WAXS mainly reveals bulk information. In the past years, a few works investigated surface reactivity by studying the reoxidation of the reduced metal surfaces.^34,80−82^ For instance, a study of Co-based surfaces by CV showed that the electrochemical oxidation of Co^2+^ to Co^3+^ occurred earlier on a surface containing more defects.^82^ The presence of defects can facilitate OH^–^ adsorption and dissociation at the surface, and results in an easier diffusion of oxygen into the bulk structure. For Cu-based catalysts, the analysis of well-defined Cu surfaces by CV has shown that *OH and *O adsorptions occur at different potentials for the (110), (111), and (100) facets, indicative of different reactivities between the facets.^83^ The simultaneous presence of multiple facets and defects in polycrystalline Cu nanoparticles typically complicates the analysis of CVs in the *OH/*O adsorption fingerprint region (+0.2 to +0.5 V *vs* RHE).^83^ Yet, the presence of defects and certain surface orientations can make the diffusion of oxygen atoms into the surface easier.^34,80−82,84,85^ Studying bulk oxidation can, therefore, be used as a proxy to characterize more reactive surface structures. In thermal oxidation, O_2_ chemisorption and O^–^ diffusion into Cu (110) surfaces are promoted by step edges compared to Cu (111) and (100) surfaces.^85^ Hirsimäki and Chorkendorff also concluded that under-coordinated sites enhance O_2_ dissociation. In addition, simulations of the Cu-water interface showed that under-coordinated sites increased the density of water molecules at the surface.^86^ Finally, a combined HERFD-XAS and operando STEM study demonstrated that grain boundary-rich Cu particles were more significantly oxidized upon air exposure after CO2RR than nondefective particles.^34^ Therefore, specific structures and defects facilitate the dissociation of O-containing species (H_2_O, OH^–^, O_2_) and the diffusion of O^–^ into the surface.

As described in the introduction of this work, the formation of C_2+_ products is enhanced by under-coordinated surface Cu sites.^34^ Yet, a too-low coordination number of the surface atoms promotes instead the reduction of water to H_2_ (HER), thus decreasing the formation of C_2+_ products from CO2RR.^36^ This implies that a high density of under-coordinated sites having intermediate coordination number might be best suited to reach high CO2RR activities and FE to C_2+_ products.

**Figure 4:**
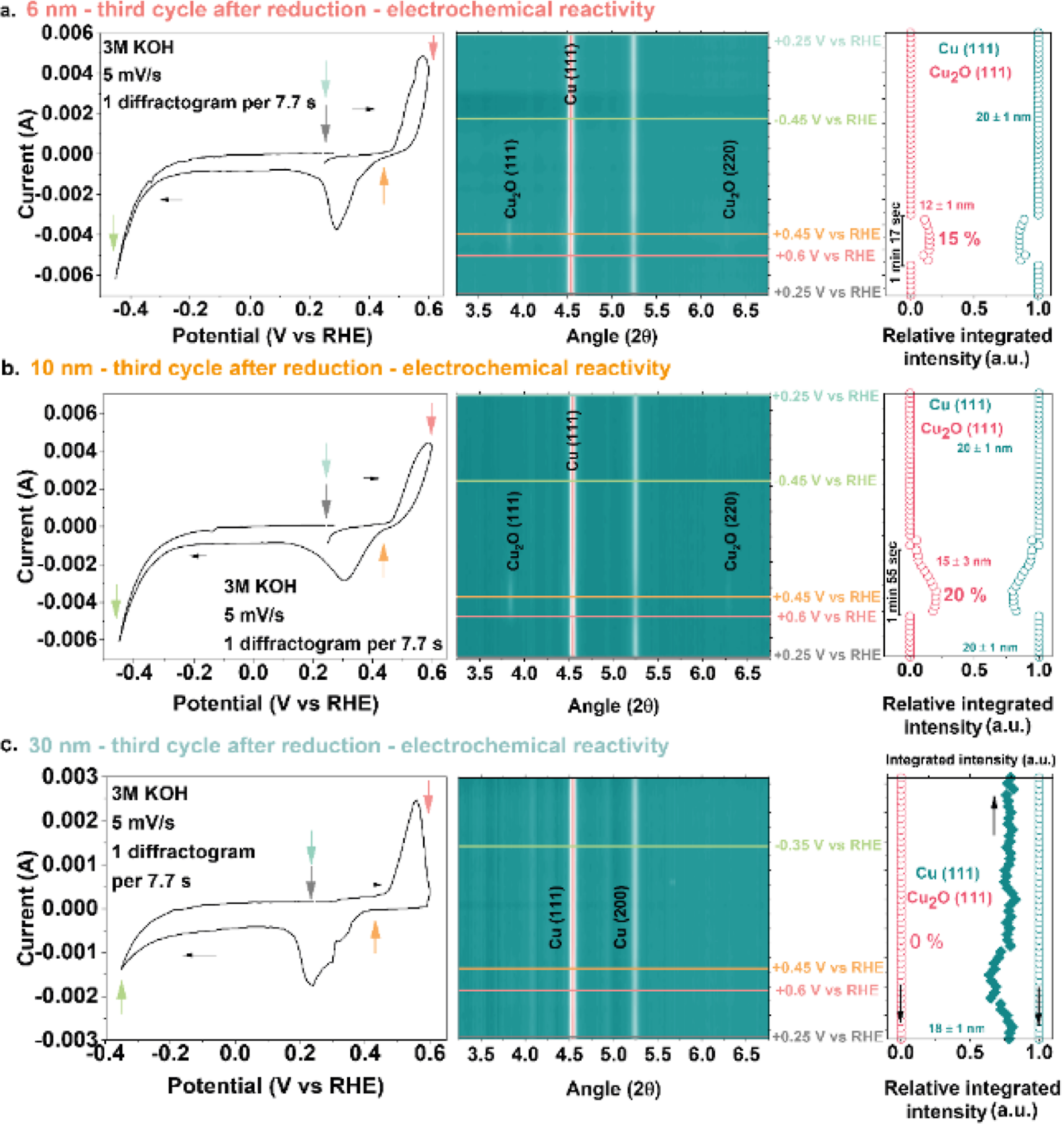
Electrochemical reactivity followed by WAXS. (a) Third CV cycle of the 6 nm CuO particles, electrochemical reactivity experiments, (b) third CV cycle of the 10 nm CuO particles, electrochemical reactivity experiments, (c) third CV cycle of the 30 nm CuO particles, electrochemical reactivity experiments.

In our work, Cu particles with similar particle and crystallite sizes were obtained from the electrochemical reduction of CuO precursors with initially different sizes. Despite their similar bulk descriptors, these Cu particles demonstrated different CORR performance. We hypothesize that, despite their similar sizes, these Cu particles may contain different surface topologies responsible for their CORR performance. As observed in the previous section, these distinct surface topologies likely arise from different Cu formation rates during the reduction of the CuO precursors of different particle sizes. Accordingly, we studied the surface structures of these reduced Cu particles by electrochemical oxidation during CV in combination with *in situ* WAXS characterization (Figures 4 and S27–S33). These measurements were carried out *in situ* and directly after the CA measurements presented in the previous section (Figures 3f, S18, S20, and S22). This implies that the Cu particles, obtained from the reduction of 4, 6, 10, and 30 nm CuO precursors during CA, were fully reduced and composed of particles with an average particle size of ∼30 nm and a crystallite size of ∼17 nm. For simplicity, we will refer to these samples using the initial CuO particle sizes, namely 4, 6, 10, and 30 nm.

It is also important to note that the electrodes remained in the cell between the CA and the electrochemical oxidation measurements, meaning they were not exposed to air. The CV cycling was carried out in the +0.6 to −0.35 and +0.6 to −0.45 V *vs* RHE ranges, while WAXS patterns were simultaneously recorded. This way, information originating from the surface and the bulk of the catalysts could be collected.

In the first CV cycle (Figures S27, S29, S30, and S31a), Cu-oxidation to Cu_2_O during the anodic sweep occurred at different potentials for the various samples.^87^ The oxidation of Cu to Cu_2_O started at +0.37 V *vs* RHE for the 4 nm sample (Figures S27 and S33). While this oxidation event was observed from +0.45 V *vs* RHE onward for the 6 and 10 nm samples, it started at a slightly higher potential of +0.47 V *vs* RHE for the 30 nm sample (Figures S29, S30, S31a, and S33). Oxidation events starting at a lower potential can be related to more defective surfaces, facilitating OH^–^ adsorption and dissociation, followed by O^–^ diffusion in the Cu lattice. Thus, the different potentials of Cu-oxidation indicated that the surface defect density on the Cu particles decreased in the order 4 nm > 6 and 10 nm > 30 nm. In the corresponding WAXS patterns, the (111) and (220) reflections of Cu_2_O, corresponding to bulk oxidation,^76^ appeared at a potential of +0.6 V *vs* RHE. No CuO reflections were observed in these CV-WAXS measurements. Once the Cu_2_O-to-Cu reduction potential was reached (between +0.37 and +0.45 V *vs* RHE) during the cathodic sweep, the Cu2O reflections disappeared from the WAXS patterns.

The phase evolution was estimated by calculating the integrated intensity (areas) of Cu_2_O (111) and Cu (111) and their relative integrated intensity (Figures S29, S30, S31a, and S33). During oxidation, the relative integrated intensity of Cu_2_O (111) reached a maximum of 18, 24, 28, and 27% for the 4, 6, 10, and 30 nm samples, respectively. Thus, the Cu-oxidation degree was the highest for the 10 nm sample. The crystallite size of the Cu_2_O phase, estimated using the Cu_2_O (111) reflection, was the same for all samples, *i.e.*, ∼ 16 nm. Although the magnitude of Cu-oxidation and Cu_2_O reduction currents were in the same range for all samples, the potential range over which the Cu_2_O phase was visible in the diffractogram differed. The duration during which Cu_2_O was present in the WAXS patterns corresponds to a certain potential range, considering that the scan rate was 5 mV/s in all cases. While the Cu_2_O phase was visible for ∼120 s in the WAXS patterns of the 6 nm sample, it remained visible for ∼150 s in the 30 nm sample and for ∼170 s for the 10 nm sample (Figures S29, S30, S31a, and S33). Therefore, the electrochemical oxidation of the 10 nm sample was the most prominent in duration and intensity among the samples evaluated. This can point to the high density and stability of the under-coordinated surface sites on the Cu particles derived from the 10 nm sample, facilitating O^–^ diffusion. Yet, the earlier onset of Cu-oxidation potential on the 4 nm sample indicated that its surface was likely the most defective, while the 10 nm sample was next.

The oxidation of the surface likely causes the formation of a different surface topology compared to the state of the sample after CA. Therefore, it is reasonable to state that only the first CV can be used to compare the samples. Nevertheless, the particles were not fully oxidized during the first CV, meaning that some defects propagating to the bulk (*e.g.*, dislocations, stacking faults, *etc.*) can be preserved. Therefore, we also carried out subsequent CVs in combination with WAXS to probe the stability of the defective structures.

During the second and third CV cycles, the 6 and 10 nm samples were less susceptible to oxidation as the relative integrated intensity of Cu_2_O (111) and the duration over which Cu_2_O was visible in the WAXS patterns decreased compared to the first CV (Figures 4a,b and S29, S30). Nevertheless, potential cycling impacted less the 10 nm sample than the 6 nm one. The crystallite size of the Cu_2_O phase was constant for the 10 nm sample, while it decreased for the 6 nm one. The behavior of the initially 4 nm sample resembled that of the 6 nm one (Figures S27 and S28). Despite oxidation currents being measured for the 30 nm sample in the second, third, and fourth CV cycles, Cu_2_O was not observed in the WAXS patterns despite the decrease in the integrated intensity of the Cu (111) reflection (Figures 4c and S31b, S32). The formation of a (disordered) surface passivation layer that prevents bulk oxidation can explain the absence of Cu_2_O/CuO reflections during consecutive CV cycling.^88^ As the TEM images of the used 30 nm sample (Figure 3a) showed that the particle size of this sample did not change much upon CORR, it might be that the restructuring during reduction was minimal for this sample. This limited restructuring may also lead to a lower density of under-coordinated Cu sites, aligning with the weak response to electrochemical oxidation. In contrast, despite reaching a similar size upon CORR, the 10 nm sample showed a greater response to oxidation. The restructuring of this sample, as seen by TEM and confirmed by *in situ* WAXS, may have resulted in the formation of more under-coordinated surface sites. Furthermore, we speculate that the surface structures grown by slow restructuring are less defective and, hence, display a lower reactivity than those grown rapidly. Our data indicates that restructuring of the 4 and 6 nm samples was much faster than that of the 10 nm sample. Structures with higher coordination than the ones of the 4 nm sample can be expected to show enhanced stability, explaining the reproducible response to oxidizing potential of the 10 nm sample. On the contrary, the 4 and 6 nm CuO precursors underwent quick and substantial structural changes in the first CuO reduction cycle to Cu (previous section), as evidenced by *in situ* WAXS. The hypothesis that catalyst restructuring leads to more defective surfaces has been postulated before.^89−91^ Oxidation of the 4 nm sample started at a lower potential than the other samples, confirming that this sample contains the most defective surface. The high reactivity of the surface of the 4 nm sample limits its stability,^70^ resulting in a rapid decrease of the initially strong oxidation features for this sample.

It has been postulated before that differences in surface atom arrangements can explain the different responses to oxidation of Cu-based nanoparticles, where defective surfaces promote O^–^ binding and diffusion.^34^ Moreover, different surface atom arrangements are responsible for steering the selectivity of CORR toward H_2_ or C_2+_ products. Specifically, defective sites were correlated to strong oxygen binding properties, stabilizing CH_2_=*CH–*O intermediates leading to acetate and ethanol.^57,60,92^ Yet, the under-coordination of these sites promotes H_2_ formation.^35^ Therefore, the presence of surface Cu atoms with intermediate coordination numbers appears important for producing C_2+_ products and suppressing HER.^49,93,94^ The electrochemical oxidation experiments revealed that the 10 nm sample contained more under-coordinated sites than the 30 nm sample, while the 4 nm sample contained the most defective surface. Yet, the response of the 10 nm samples was more stable than that of the 4 and 6 nm samples during subsequent CV cycles. We tentatively attributed these differences to the density and the type of defects at the reduced Cu surface. The intermediate reactivity and high stability of the 10 nm samples during CV cycling might indicate that its surface contains Cu surface atoms with intermediate coordination number, which are better retained during oxidation–reduction cycles than less coordinated Cu surface atoms on the 4 and 6 nm samples. Simultaneously, we observed that H_2_ formation is promoted on the 4 nm sample, correlating with the highly defective nature of the surface. C_2+_ oxygenates are, instead, formed primarily on the 10 nm sample, which contains defects with higher apparent stability, with higher atom coordination numbers or of different nature (*e.g.*, dislocations, stacking faults). The weak response to oxidation of the 30 nm sample suggests fewer under-coordinated sites with more saturated Cu surface atoms than in the 10 nm sample, resulting in a lower FE to C_2+_ oxygenates.

**Figure 5:**
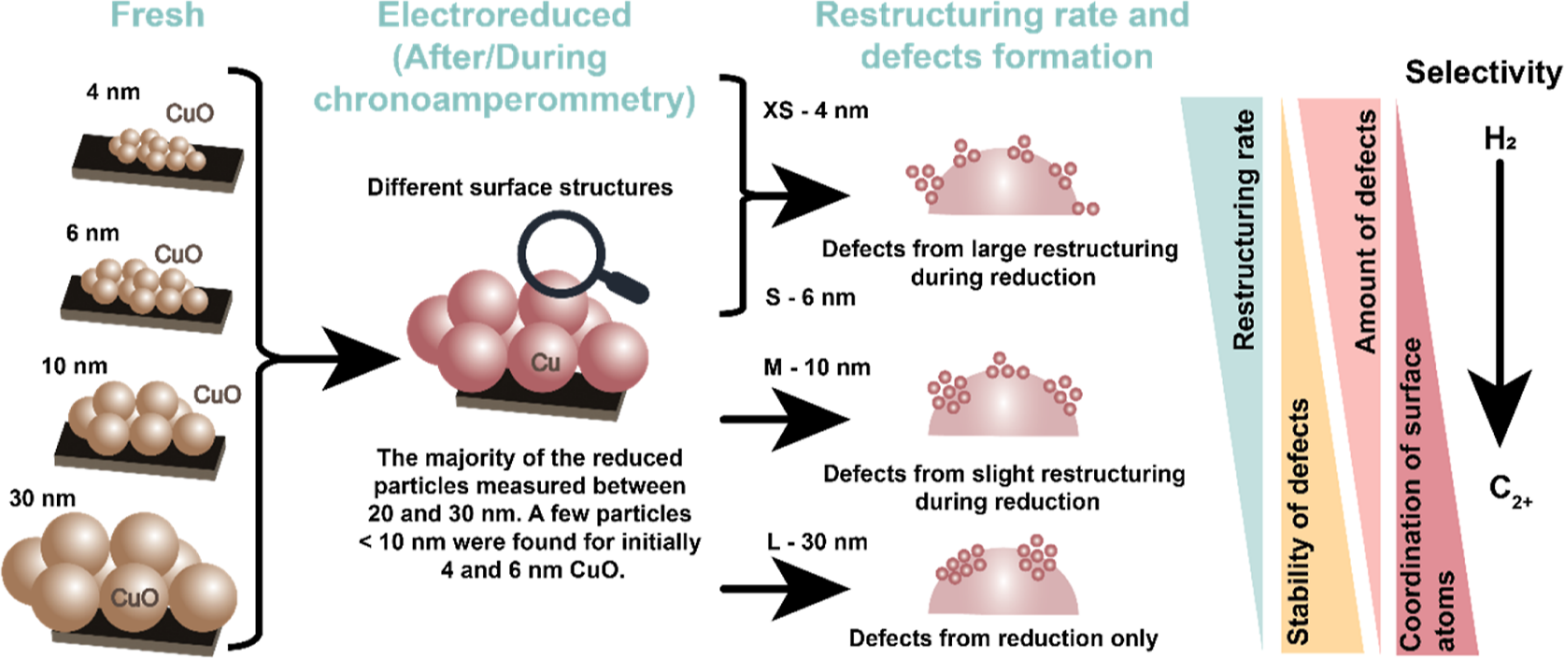
Structural transformation from the fresh CuO nanoparticle state to the reduced Cu nanoparticle state, highlighting the defect formation during the restructuring of the CuO phases under CORR conditions.

Although these data do not allow us to draw a quantitative picture of the surface structures formed during reduction, our results provide indications that differences in density and reactivity of defects can explain the catalytic trends for CORR over similarly sized Cu particles, which originate from the reduction of CuO particles of different sizes (Figure 5). Our study emphasizes the need to characterize Cu catalysts after and, preferably, during electrochemical reactions. Besides an intrinsic effect of the initial CuO particle size on the final particle topology, the electrochemical pretreatment strongly affects the surface structures, impacting the overall reactivity and stability by forming various defects. We, therefore, believe that investigating the impact of the electrochemical pretreatment (CV potential range, scan rate, electrolyte, pulses, *etc.*) on the as-synthesized catalysts is crucial to modulate the structure of Cu-based catalysts to enhance the selectivity to C_2+_ products. Finally, while our work focused on the assessment of the structural transformation of the catalysts during the CV pretreatment and the structure-selectivity relationship in CORR, we suggest that additional research on the CORR reaction mechanism would help establish the relationship between defect density/types and C_2+_ reaction pathways on catalysts derived from oxidized precursors.

## Conclusion

CuO particles of different sizes (4–30 nm) were obstained by FSP and their performance in the CORR to C_2+_ products were evaluated. After CuO reduction to Cu under CORR conditions, the particle size (∼30 nm) and the crystallite size (∼17 nm) of the Cu particles were similar for all samples, regardless of the initial CuO size. Despite this, the samples presented different FEs to C_2+_ products. Particles derived from very small (4 nm) and small (6 nm) CuO precursors displayed a high FE toward H_2_, while particles derived from larger CuO samples (≥10 nm) favored C_2+_ formation. The highest C_2+_ product FE (60%) was obtained for the sample derived from the 20 nm CuO precursors. Following the structural changes of the initial CuO precursors during reduction by *in situ* characterization, we could establish that all samples were reduced to metallic Cu with negligible amounts of Cu^+^/Cu^2+^. As the main bulk descriptors (phase, particle and crystallite size) were the same for the samples during CORR, we surmised that the Cu particles of similar size exposed different surface structures. These differences are likely due to the restructuring taking place during the reduction of CuO to Cu, which may depend on the size of the initial CuO precursors. Electrochemical oxidation of the reduced Cu particles after CORR, followed by CV coupled with WAXS, confirmed that the propensity of surface oxidation, which can be linked to the under-coordination of surface atoms, decreased in the order 4 nm CuO > 6 and 10 nm CuO > 30 nm CuO. These surface oxidation events preceded bulk oxidation, gauged by *in situ* WAXS. Bulk oxidation of the Cu particles derived from the 10 nm sample was the most intense and stable during CV cycling, which indicated the more disordered nature of the surface compared to the less defective surface of the 30 nm sample and the higher coordination number of the surface atoms compared to the more reactive 4 and 6 nm samples.

The significant oxidation of the 4 and 6 nm samples in the first CV eroded quickly during subsequent CV cycles, likely caused by the stronger under-coordination of the surface of these samples compared to the 10 nm sample. The differences in the density and reactivity of the under-coordinated surface atoms provide a qualitative explanation for the observed selectivity differences, as it is known that strongly under-coordinated Cu sites facilitate HER. Less under-coordinated atoms (having an intermediate coordination number) are necessary to favor *CO–*CO dimerization, which can explain the optimum performance of the 10 and 20 nm samples. The substantial restructuring of the CuO precursor into Cu particles of similar size during reduction depends on the initial CuO size and leads to different surface structures. These surface structures affect the product distribution in CORR by enhancing either *CO–*CO dimerization or H_2_ evolution as a function of coordination of the surface atoms.
