# Supplementary material for: Restructuring of Cu-based Catalysts during CO Electroreduction: Evidence for the Dominant Role of Surface Defects on the C2+ Product Selectivity
Source: ACS Catal. 2024 Aug 20;14(17):13246–59. doi: 10.1021/acscatal.4c02718 (PMC11385435; doi:10.1021/acscatal.4c02718)
Supplement: Supplementary file 1 — cs4c02718_si_001.pdf [file cs4c02718_si_001.pdf]

Supplementary information

Floriane A. Rollier, Valery Muravev, Alexander Parastaev, Rim C.J. van de Poll, Jason M.J.J. Heinrichs, Bianca Ligt, Jérôme F.M. Simons, Marta Costa Figueiredo, Emiel J.M. Hensen*

Laboratory of Inorganic Materials and Catalysis, Department of Chemical Engineering and Chemistry, Eindhoven University of Technology P.O. Box 513, 5600 MB Eindhoven (The Netherlands)

*Corresponding author: Prof. Emiel Hensen, e.j.m.hensen@tue.nl

Methods and Materials – additional details

Chemicals

$Cu(NO_3)_2.3H_2O$ 99% was purchased from ACROS organics. Absolute ethanol (extra-dry) was supplied by Biosolve B.V. 2-ethylhexanoic acid (99%), and potassium hydroxide solution 47 wt% for analysis EMSURE were purchased from Merck Life Science NV. Carbon paper (SIGRACET 22BB, Ion Power GmbH) and anion exchange membrane (Fumasep FAA-3-PK-130, Fumatech BWT GmbH) were used as Gas Diffusion Layer (GDL) and membrane, respectively. Nafion 5 wt. % suspension (D-521) in water and light alcohols was obtained from Merck Life Science NV.

For ^{1}H-NMR analysis, DMSO (99.9% - AR grade – Biosolve), phenol (99.5% - Merck), and D_2O (99.9% - Sigma Aldrich) were used to prepare the internal standard, as later described. External calibration was prepared using analytes obtained from different suppliers (Methanol 99.9%, ethanol 99.9%, propanol 99.9%, formic acid 98%, acetic acid 99.7%, propionic acid 99%, and acetaldehyde 99.5%). Milli-Q water was used for all the experiments and obtained from an Elga Purelab flex system.

Ex situ characterization

Lab-based X-ray diffraction (XRD) was used to analyze the crystalline structure of the samples. A Bruker D2 phaser diffractometer and a Cu Kα X-ray (0.15406 nm) source were used.

(Quasi) in situ characterization

Quasi-in situ X-ray photoelectron spectroscopy was used to study the oxidation state and surface composition during/after reduction. The measurements were carried out on a SPECS system. An ambient pressure electrochemical cell attached to the system allows to perform electrochemical reactions in an inert atmosphere. The working electrode was prepared as described in the "electrode preparation" section. A Reversible Hydrogen Electrode (RHE, miniRHE Gaskatel) and a Pt foil were used as reference and counter electrodes, respectively. A 3 M KOH solution was used as electrolyte and was purged with He for 20 min before any electrochemical experiments to ensure oxygen removal. Afterwards, the He flow was kept constant until the transfer of the sample in vacuum was completed. A cyclic voltammetry (CV) measurement, from +0.5 V vs RHE to -1.0 V vs RHE, was recorded at a scan rate of 50 mV/s. The electrode was then rinsed six times with Ar-purged ultrapure water to remove the remaining KOH. Once all the liquid was removed, the transfer from the electrochemical cell to the buffer chamber was carried out under He atmosphere ensuring an air-free transfer. The buffer chamber was then evacuated until high vacuum was reached. Next, the sample was transferred into the analysis chamber with a base pressure below 10^{-9} mbar. Similarly, chronoamperometry (CA) measurements were carried out at -0.5V vs RHE until a specific charge was reached, -0.5 C and -13.0 C. A new fresh electrode was used for each of the CV and CA measurements. For the XPS measurements, a monochromated Al Kα source was used at 50 W. Cu 2p, Cu LMM, O 1s, C 1s, and survey spectra were recorded for each sample. The binding energies were corrected on the C-C component of the C 1s spectra (284.5 eV). The fitting parameters related to Cu species were taken from the literature [1]. First, a Shirley background was subtracted. The Cu $2p_{3/2}$ was fitted using a GL (30) function for CuO and $Cu(OH)_2$ components while GL (80) was used for $Cu^{+/0}$. The Cu LMM was analyzed based on the maximum kinetic energy.

In situ wide-angle X-ray scattering (WAXS) measurements were performed at the beamline ID31 of the ESRF synchrotron radiation facility. The incident photon energy of 75 keV (0.0165 nm) and a Pilatus CdTe 2M detector were used in a Debye-Scherrer geometry (Transmission configuration). For these measurements, a homemade electrochemical cell was developed (Fig. S35). Pt wires were used as pseudo-reference (Qref) and counter

electrodes respectively. The working electrode was composed of a catalyst layer drop-casted on carbon paper, as described previously. PEEK windows of 250 µm thickness were used to minimize X-ray absorption. The He-flushed electrolyte (3 M KOH) was flown through the cell during the experiments. The conversion step from the pseudo-reference electrode (Qref) to the RHE scale was determined by measuring a cyclic voltammogram, where the onset of *OH/*O adsorption on Cu surfaces was found at -0.55 V vs Qref. From previous CVs, the *OH/*O adsorption was seen at +0.35 V vs RHE. Therefore, we convert the potential using the following expression:

$E_{RHE} = E_{Qref} + 0.9$ V

At first, a cyclic voltammetry measurement was carried out between +0.40 V vs RHE and -0.35 V vs RHE using a 5 mV/s scan rate. In total, 6 cycles were recorded to probe the phase changes and crystallite sizes during the pretreatment, i.e., cyclic voltammetry. A chronoamperometry measurement at -0.3 V vs RHE was then started and a similar charge was spent for all measured samples. Afterward, probing the electrochemical oxidation of the reduced catalysts was done by extending the range of the CV to more oxidizing and reducing potentials (from +0.60 V vs RHE to -0.35V vs RHE (1) and -0.45 V vs RHE (2), scan rate: 5 mV/s). WAXS was recorded during all electrochemical measurements.

The data treatment consisted in first applying a background subtraction to the recorded diffractograms. An ink containing only Nafion and ethanol, in identical proportions as in a catalyst ink, was deposited on carbon paper. This carbon paper was placed in the in situ WAXS cell with flowing electrolyte and the resulting diffractogram was used as background. The transfer of the reflection position from q (reciprocal space) to 2θ (real space) space was calculated using the following formula: $|q| = \left(4 * \frac{\pi}{\lambda}\right) * \sin\left(\frac{2\theta}{2}\right)$. Assignment of the reflections, based on existing literature, was evaluated using Bragg's law: $n\lambda = 2d*\sin\theta$. A Voigt function was employed to fit the reflection signal presented in this study, namely CuO (111), CuO (002), Cu (111), and Cu_2O (111).

During WAXS and XPS experiments, no iR compensation was applied to the potential.

Electrochemical methods

A home-designed H-cell with a gas diffusion electrode configuration (GDE) was used during all electrochemical measurements. During all experiments, a 3 M KOH solution was used as electrolyte. Catholyte and anolyte were separated by an anion exchange membrane (Fumasep FAA-3-PK-130). A Pt foil and a Reversible Hydrogen Electrode (RHE, miniRHE Gaskatel) were used as counter and reference electrodes, respectively.

Cyclic-voltammetry (CV) – Cyclic-voltammograms of all the catalysts tested in this study were recorded using a potentiostat (Metrohm AUTOLAB PGSTAT302N). The electrolyte was purged for 15 min with 15 ml/min of either He or CO before experiments. The CVs were then recorded between -1.0 V and +1.5 V vs RHE. A scan rate of 50 mV/s was applied, and 5 scans were recorded. Before CORR chronoamperometry, only short-range CVs were recorded in He from -1.0 V vs RHE to +0.5 V vs RHE, 50 mV/s. The potential range was shortened to avoid oxidation of the catalyst and possible loss of material. Moreover, no CV in CO was carried out before the chronoamperometry measurements to ensure that all carbon-based products originated from CORR chronoamperometry (CA).

Electrochemical Impedance Spectroscopy (EIS) – EIS was measured for every electrode at -0.2V vs RHE. This potential was chosen as no faradaic event took place at this potential on the short-range CVs. Frequencies were ranged from 50 kHz to 0.5 Hz. 80% of the measured iR drop was used to correct the CV, the electrochemically active surface area (ECSA), and the CA measurements.

Electrochemically active surface area (ECSA) measurements (double-layer capacitance method) – Typically, the electrolyte was purged for 15 min with 15 ml/min of He before experiments. The double-layer capacitance of the electrode was then measured at different scan rates (10, 20, 50, 100, and 150 mV/s). CVs from -0.1 V to -0.2 V vs RHE were recorded as no faradaic events took place in the considered potential range. The average current of the double layer at -0.15 V vs RHE was plotted versus the scan rates. The slope gave the capacitance of the layer (Cdl) or roughness factor (RF). The ECSA itself was obtained from the following formula:

$ECSA = \frac{Cdl}{Cref}$

A C*ref* of 40 $\mu F/cm^2$ was obtained from the literature [2,3].

Catalytic performance – CORR – additional details

The GC was equipped with a FID detector for the analysis of hydrocarbons and a TCD for the detection of permanent gases (H_2 and CO). Before TCD detection, a Hayesep Q pre-column and a Shin-Carbon ST column were used to separate the permanent gases. An Al_2O_3/KCl column was used for the separation of the hydrocarbon products that were further quantified by FID. An injection was started every 8 minutes of the chronoamperometry. The constant production of gasses was monitored by the mass spectrometer while the elution took place in the GC.

The ^{1}H-NMR spectra were recorded on a (Bruker) 400 MHz spectrometer. To increase the signal-to-noise ratio, 400 scans were used. A delay time (d1) of 15 s was used to account for the spin relaxation time (T1) required by the analytes. The NMR sample was prepared by mixing 630 µl of electrolyte with 70 µl of an internal standard solution. The internal standard solution contained 10 mM of DMSO and 50 mM of Phenol in D_2O (Fig. S7).

Every measurement was run for one hour at different potentials ranging from -0.4 V vs RHE to -0.6V vs RHE. A fresh electrode was used for every single measurement. The Faradaic efficiencies were calculated using the following formula:

$$FE\ (\%) = \frac{(ne- * n\ products * F)}{Q}$$

With n_{e-} the number of exchanged electrons to produce one mol of product, $n_{product}$ the number of mol of the product calculated from GC or NMR data, F the Faraday constant (96485 C/mol), Q the charge spent over the considered unit of time (per minute or the whole measurement).

After normalization of the total current densities by the electrochemically active surface area, the partial current densities were calculated with the formula:

$$J_{partial} = FE_{product} * J_{total}$$

The average and the standard errors of three measurements were calculated.

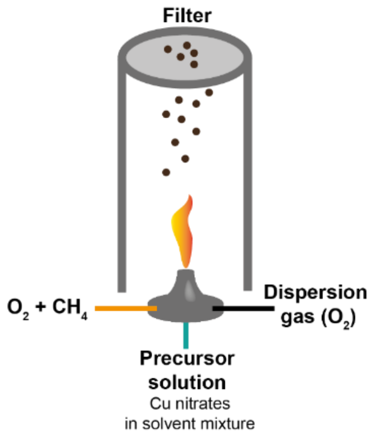

Figure S1. Flame Spray Pyrolysis schematic.

Table S1. Synthesis parameters and resulting crystallite and particle sizes.

CRYSTALLITE SIZE (WAXS) (nm)	PARTICLE SIZE (TEM) (nm)	INJECTION FLOW (ml/min)	DISPERSION FLOW (l/min)	CALCINATION
**5 ± 0.5**	4 ± 1	1	7	-
**6 ± 0.2**	6 ± 1	1	5	-
**10 ± 0.5**	10 ± 2	5	5	-
**17 ± 1**	20 ± 4	10	5	-
**20 ± 1.5**	30 ± 8	15	5	-
**25 ± 2**	70 ± 16	5	5	450°C

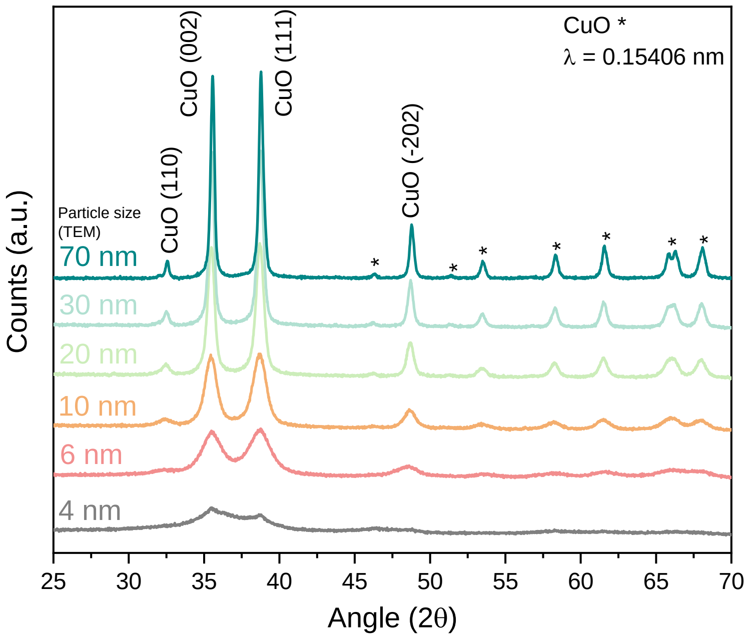

Figure S2. X-ray diffractograms of fresh catalysts.

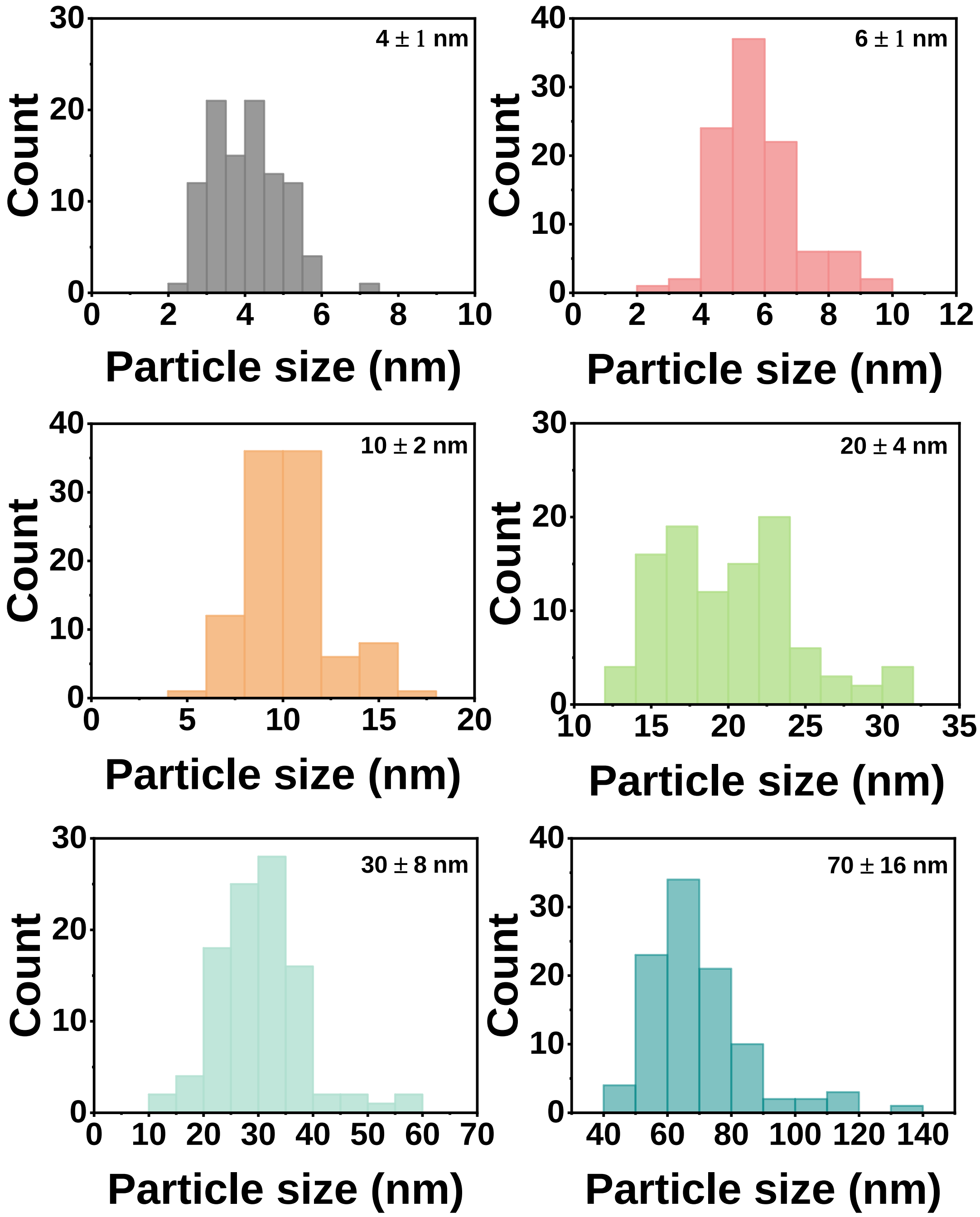

Figure S3. CuO particle size distribution measured by TEM.

Note S1.

To electrochemically probe the surface properties of prepared nanomaterials, we used cyclic voltammetry (CV). CVs were recorded in 3 M KOH using He or CO as inert or reactive gas, respectively (Fig. S4). All potentials are iR corrected by 80% of the impedance value. The different particle sizes demonstrated a similar behaviour in the HER and CORR region at potentials more negative than -0.2 V vs RHE. However, in the presence of CO, the intensity of the oxidation and reduction events of Cu species decreased significantly. At oxidizing potentials, the loss of copper at the electrode surface could be enhanced in CO-rich environment [4,5], decreasing therefore the intensity of Cu redox events. Additionally, a wide oxidation feature (Fig. S4), which was attributed to CO oxidation, appeared at an onset potential of -0.15V vs RHE [6]. The decrease in intensity of Cu redox events could be therefore caused by the adsorption of CO on the surface.

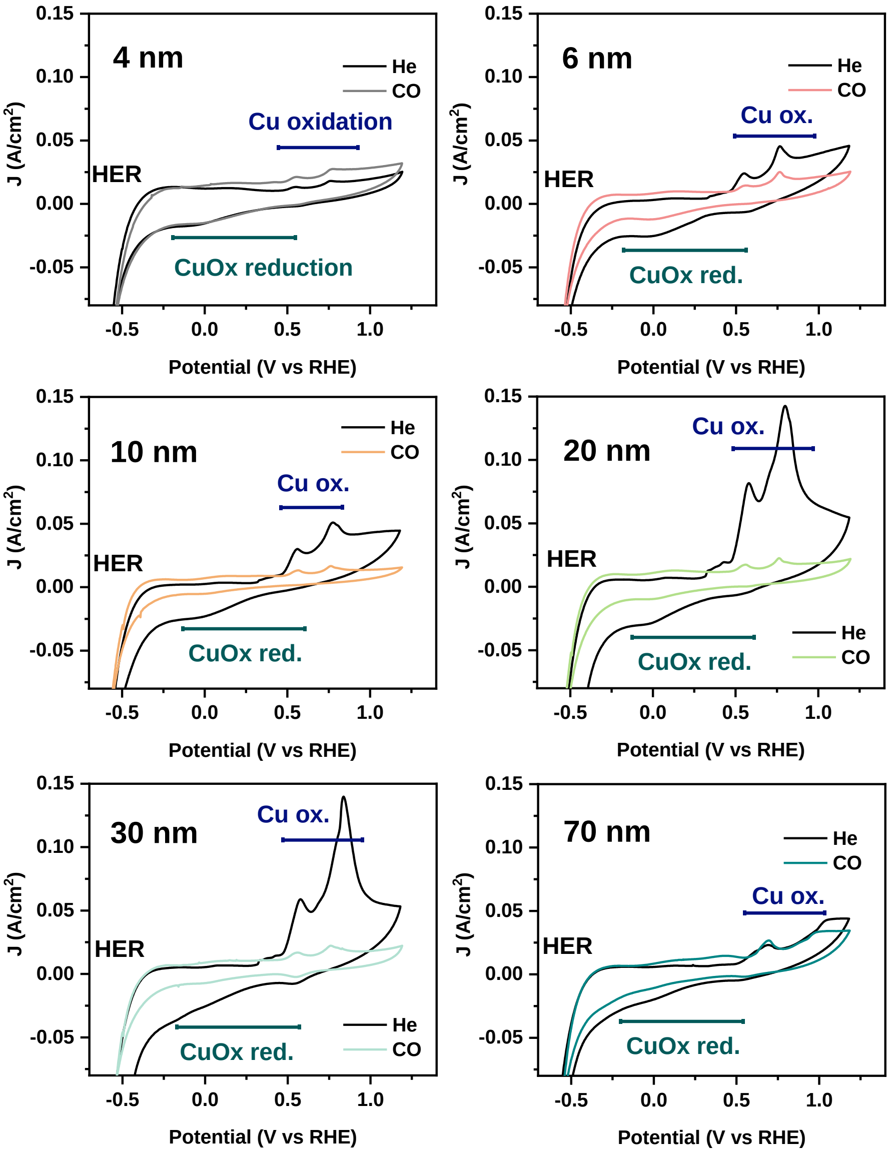

Figure S4. Cyclic-voltammograms of all samples in inert (He) or reactive (CO) atmosphere, 3M KOH, iR corrected potential – J is normalized by the electrochemically active surface area (ECSA).

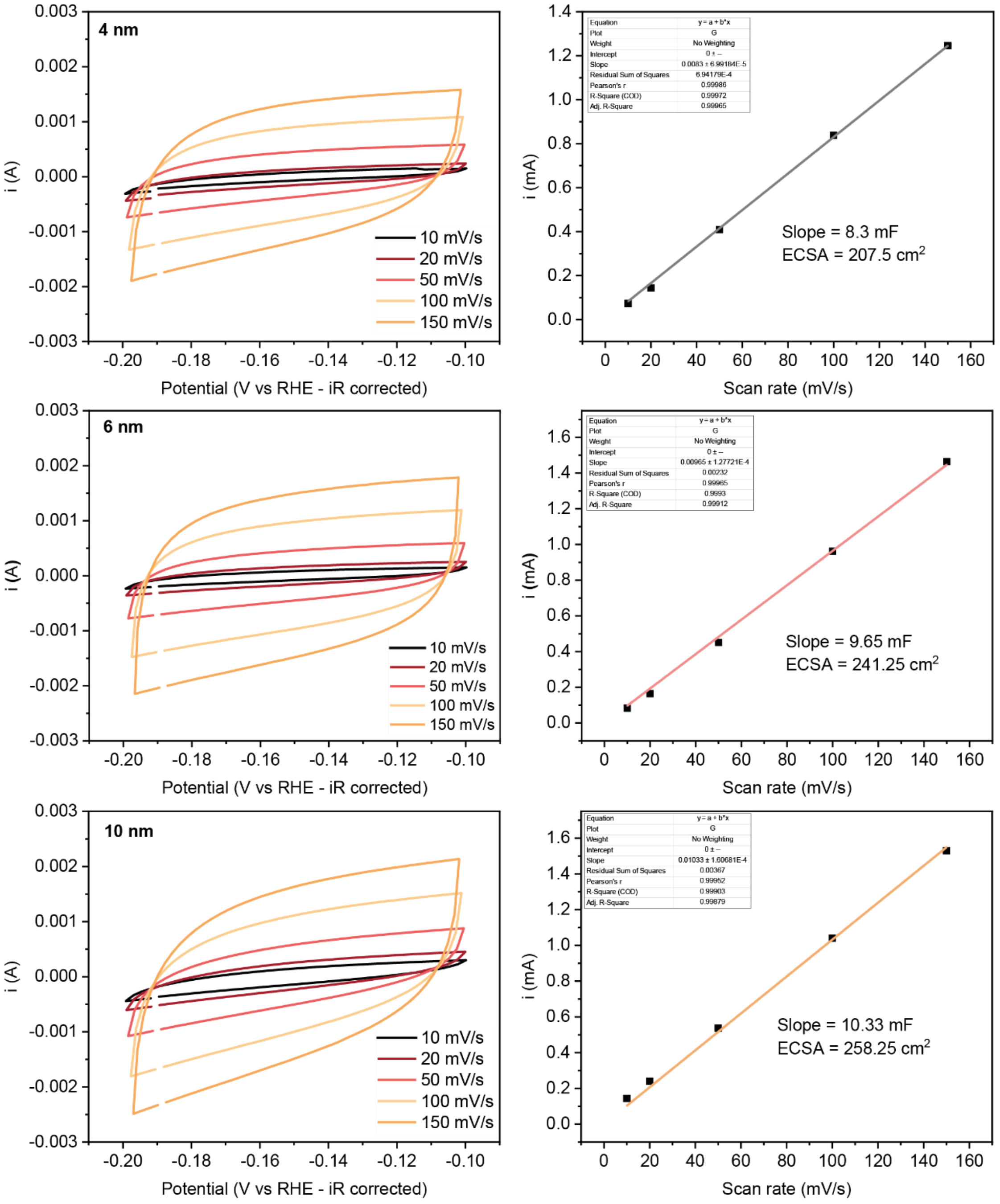

Figure S5. Electrochemically active surface area plots - one example is shown per size (1).

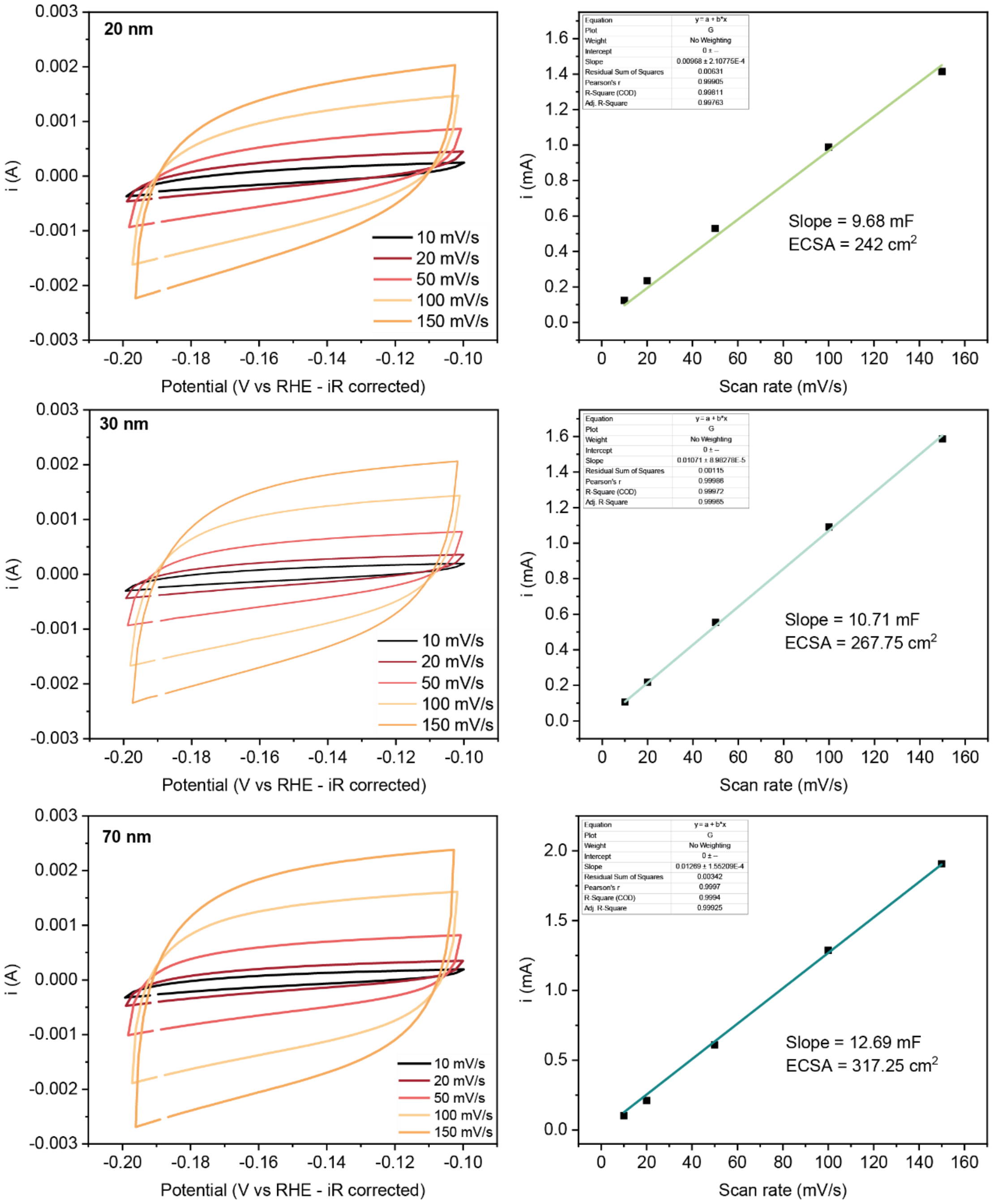

Figure S6. Electrochemically active surface area - one example if shown per size (2).

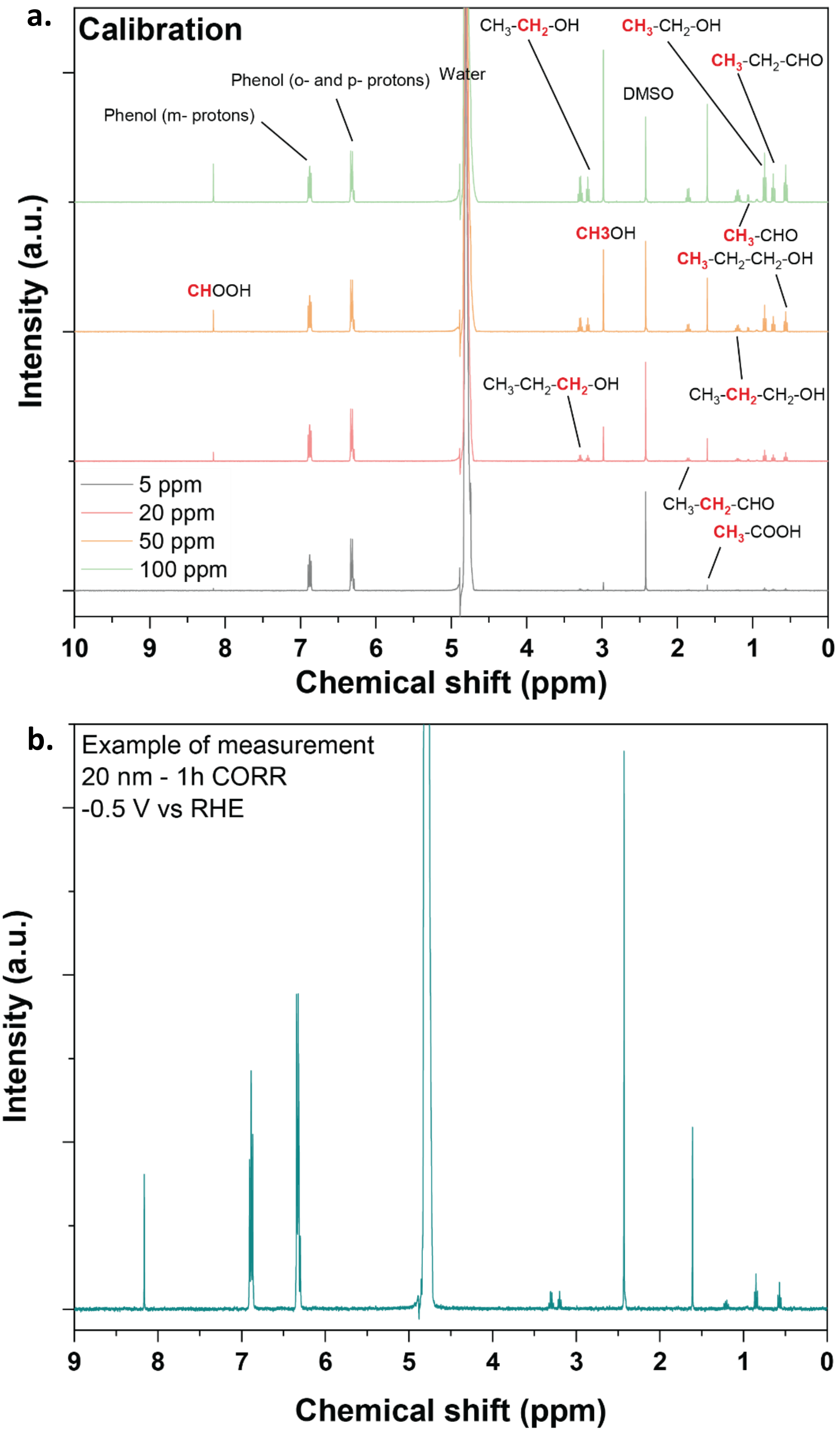

Figure S7. a) ^{1}H NMR measurement of the external calibration line (5, 20, 50, 100 ppm of analytes), b) Example of 1H NMR measurement for 20 nm sample measured at -0.5V vs RHE 1h CORR.

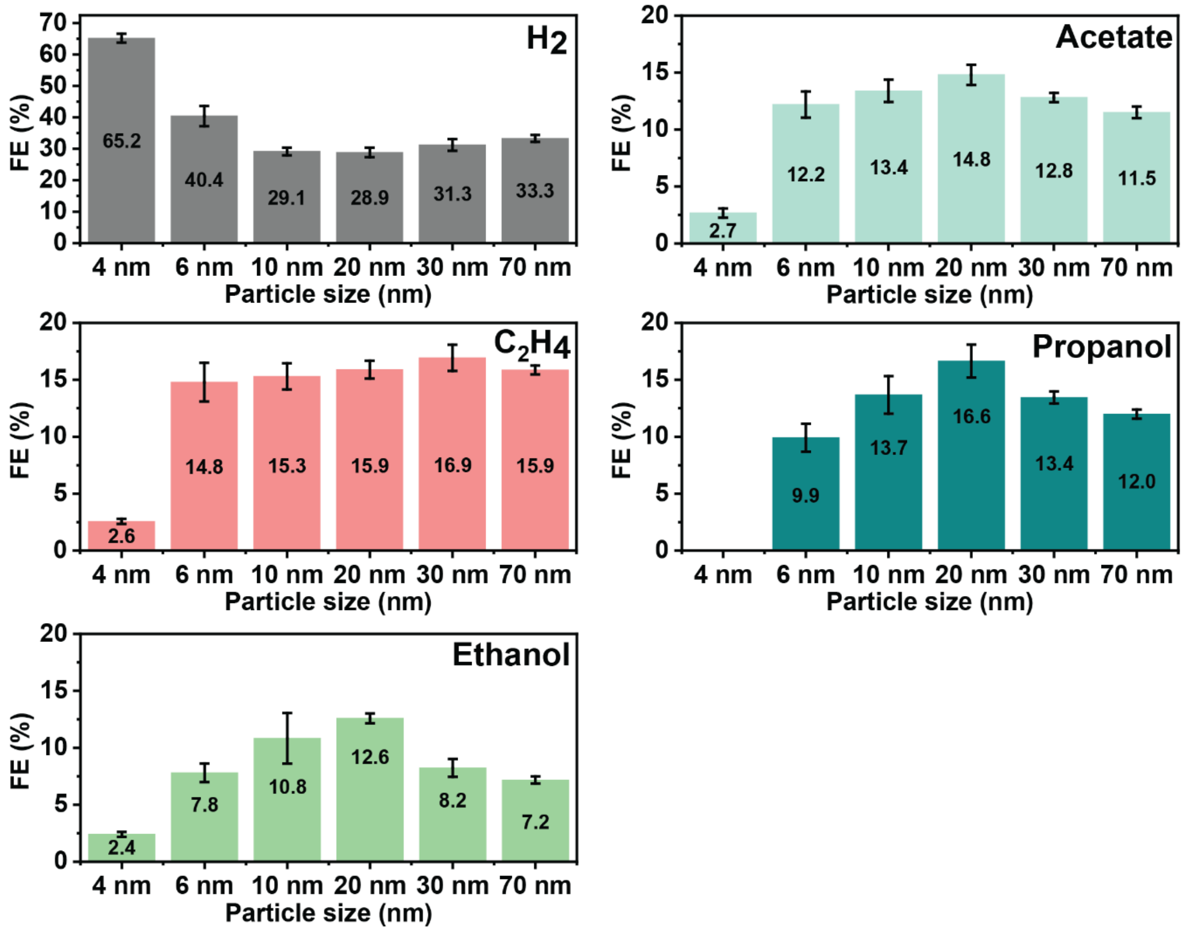

Figure S8. Faradaic efficiencies for each main product recorded at -0.5 V vs RHE for all particle sizes.

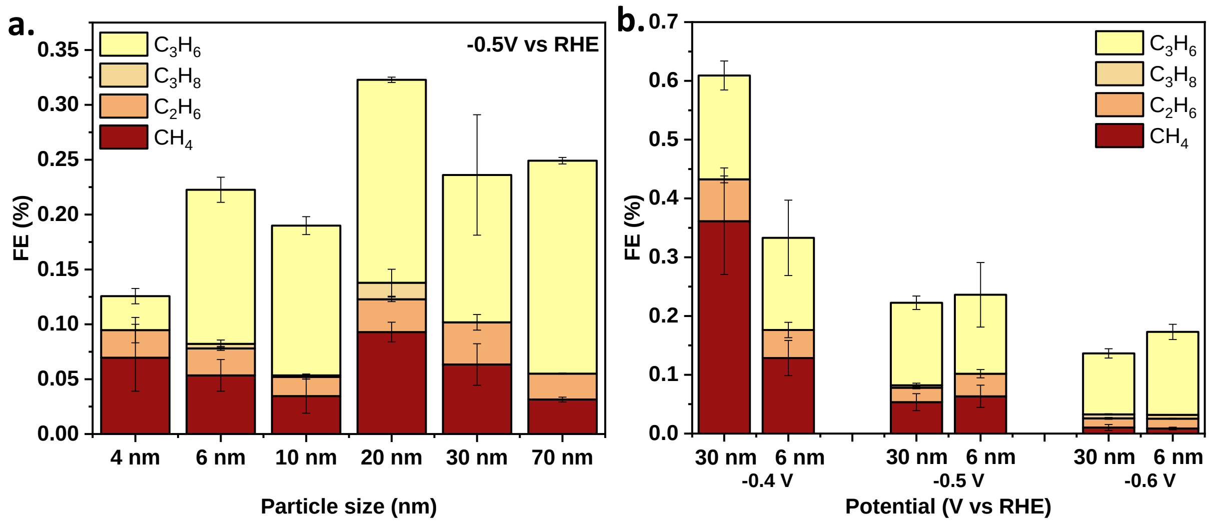

Figure S9. a) Minor side products selectivity during CORR at -0.5V vs RHE showing the particle size effect on FE, b) minor side products selectivity for the 6 nm and 30 nm sample at different applied potentials.

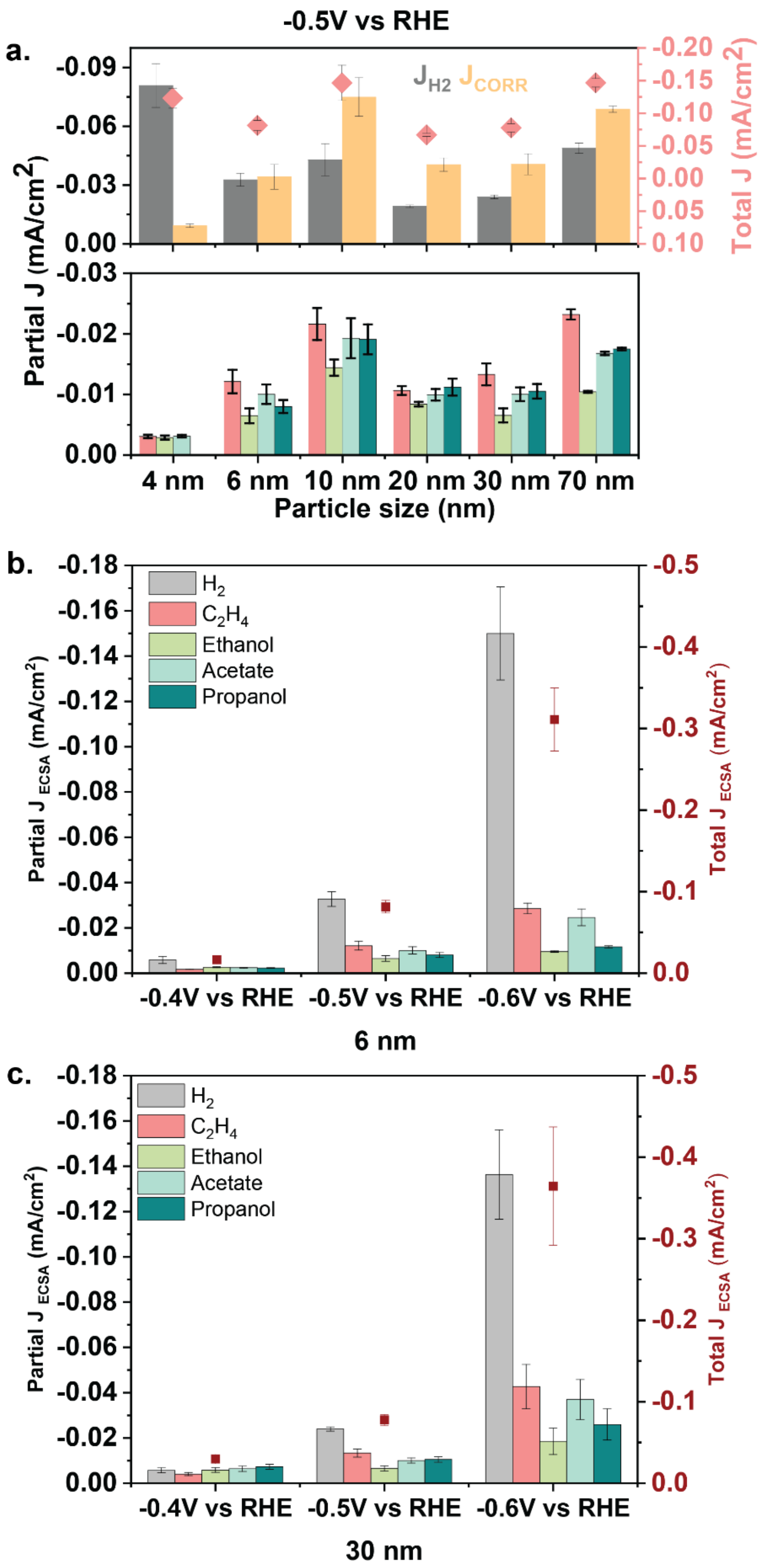

Figure S10. Normalized total and partial current densities (J) produced by a) the samples during CORR at -0.5 V vs RHE, b) 6 nm, and c) 30 nm CuO during CORR at different potentials ranging between -0.4 V vs RHE and -0.6 V vs RHE.

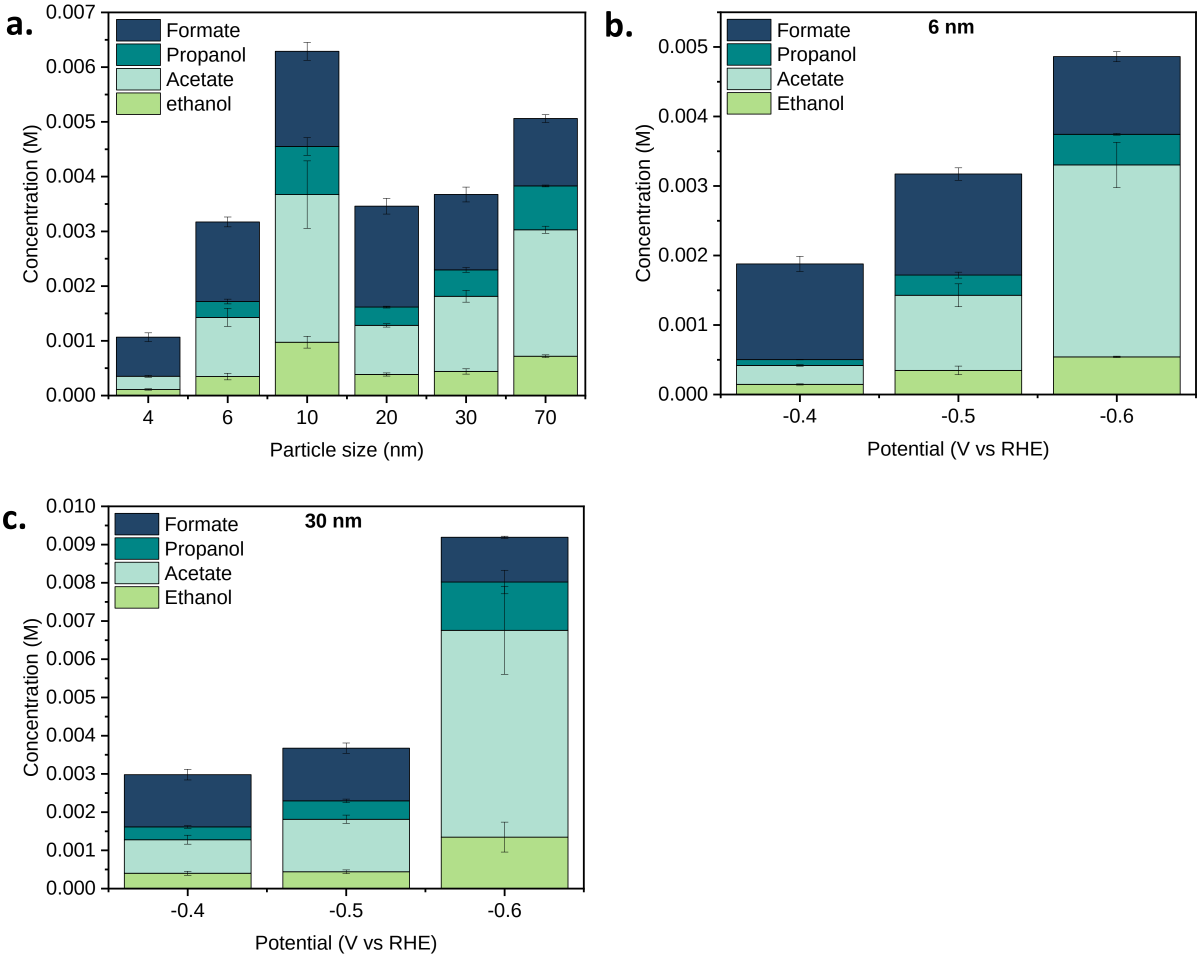

Figure S11. Concentration of liquid products in the electrolyte after reaction a) Influence of size on the concentration, b) potential effect on liquid products concentration for the 6 nm sample, c) Potential effect on the liquid products concentration for the 30 nm sample – This is especially useful to observe the final concentration of formate in the electrolyte.

Note S2. Incomplete FE and formation of formate

The decreased total FE at more negative potentials could result from catalyst restructuring (dissolution – redeposition) [7], the crossover of negatively charged products through the membrane (e.g., acetate) [8], and some unquantified minority side products. The main side products, which could be detected and quantified, are summarized in Fig. S9. Moreover, substantial amount of formate ($HCOO^-$) were unexpectedly detected. As several CO2RR mechanistic studies indicate that $HCOO^-$ and CO do not share the same reaction pathways [9], the formation of $HCOO^-$ from CO was therefore unexpected. We observed that the applied potential had a minor effect on the quantity of $HCOO^-$ byproduct formed by the 6 nm and 30 nm samples (Fig. S11b and c). However, $HCOO^-$ concentration changed with particle size (Fig. S11a). The 10 nm and 20 nm samples, producing more C_{2+} products, demonstrated a higher $HCOO^-$ concentration, while the 4 nm sample, selective to hydrogen, demonstrated the lowest $HCOO^-$ molarity.

Note S3.

The stability of the 6 nm and 30 nm samples was tested over 5 h CORR at -0.5 V vs RHE (Fig. S12). The stability tests were sequenced at 1 hour, 2.5 hours, and 5 hours in order to open the cell and collect a liquid sample. The liquid sample collected amounted to 2 ml and fresh electrolyte of the same amount was added to the catholyte chamber in order to operate at constant volume. The gaseous products were analyzed online as described in the experimental details. The catalysts showed a stable selectivity towards H_2 over the 5 h period. The selectivity towards C_{2+} products decreased from 43% to 32% for 6 nm sample and from 47% to 29% for 30 nm sample. The oxygenate selectivity was the most impacted while the ethylene FE remained constant. The drop of total FE over the tested time likely resulted from the intensification of the dissolution/redeposition process, requiring more charge to redeposit dissolved Cu ions, and the intensification of liquid products crossover through the membrane, being reinforced when the concentration of products increases in the catholyte. Over time, a decrease in the overall normalized current density (Fig. S12c and d) was also observed related to an increase in electrochemical surface area (Fig. S13). The change in surface area was observed by measuring the ECSA (double layer capacitance method) before the start of the experiment, after 1 h, after 2.5 h and after 5 h. As the amount of dissolved Cu in the electrolyte was negligeable (Table S2, below ppm level), we hypothesize that the increase of surface area is related to surface restructuring by dissolution-redeposition mechanism[7].

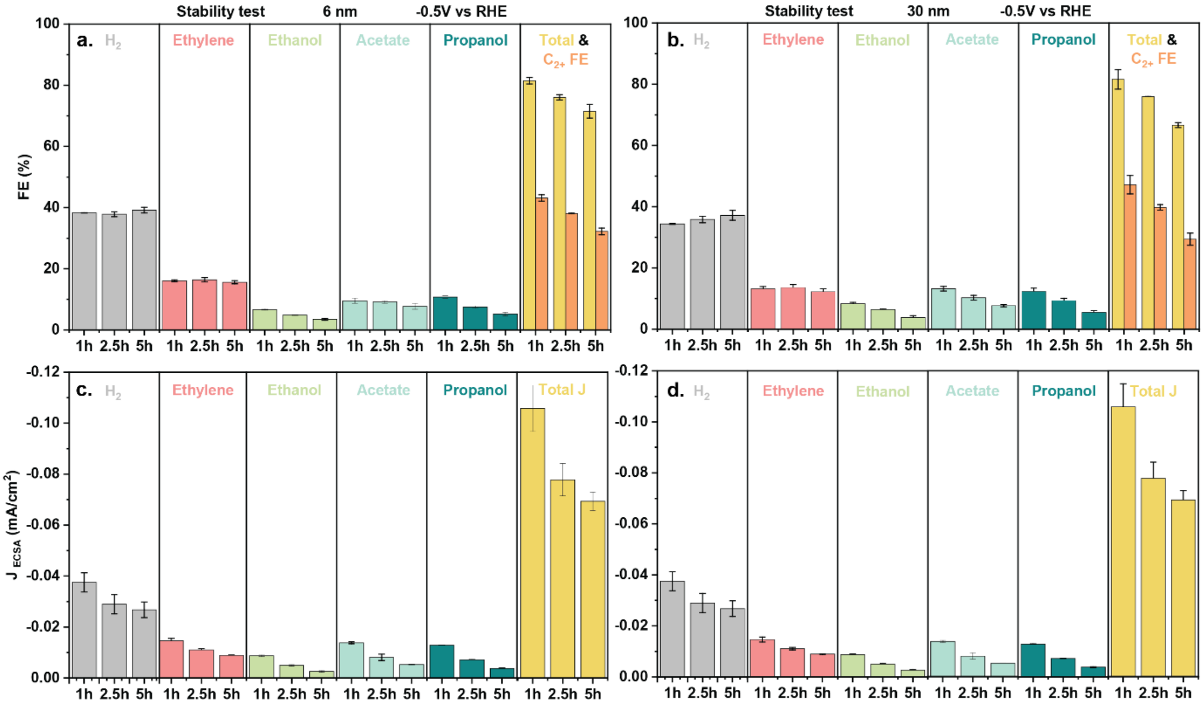

Figure S12. a) and b) Faradaic efficiencies of the major products measured over 5 h of CORR at -0.5V vs RHE on a) 6 nm and b) 30 nm CuO, c) and d) current densities of the major products measured over 5 h of CORR at -0.5V vs RHE on c) 6 nm c) and d) 30 nm CuO.

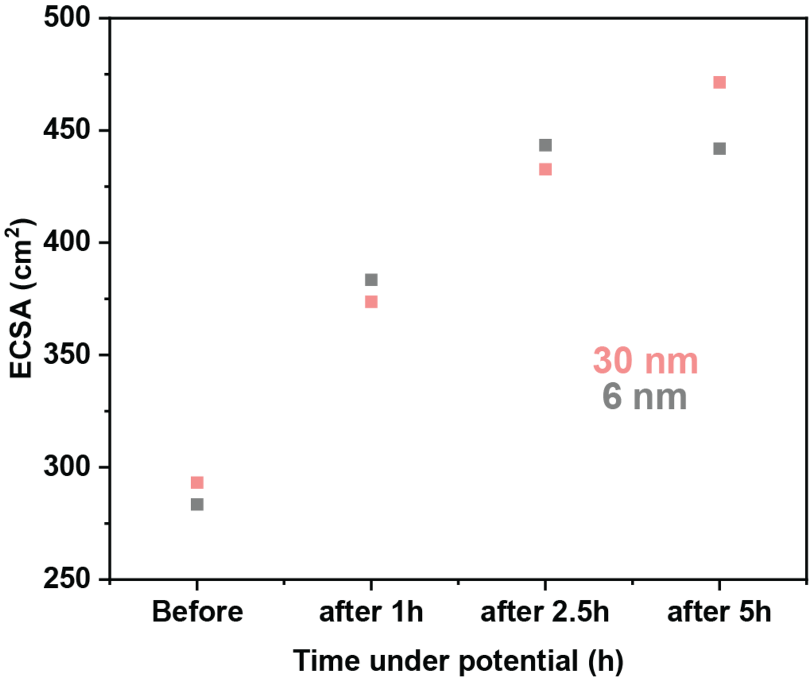

Figure S13. Evolution of the electrochemically active surface area (ECSA) over time.

Table S2. Cu content in catholyte measured by ICP-OES after a stability test of 5 h at -0.5 V vs RHE.

Stability tests -0.5V vs RHE 5h	mg/l Cu
6 nm CuO	0.02
30 nm CuO	0.06

Table S3. Cu content in catholyte measured by ICP-OES after 1 h of CORR at -0.5 V vs RHE.

Electrode at -0.5V vs RHE	mg/l Cu
4 nm	0.97
6 nm	0.37
10 nm	0.19
20 nm	0.13
30 nm	0.24

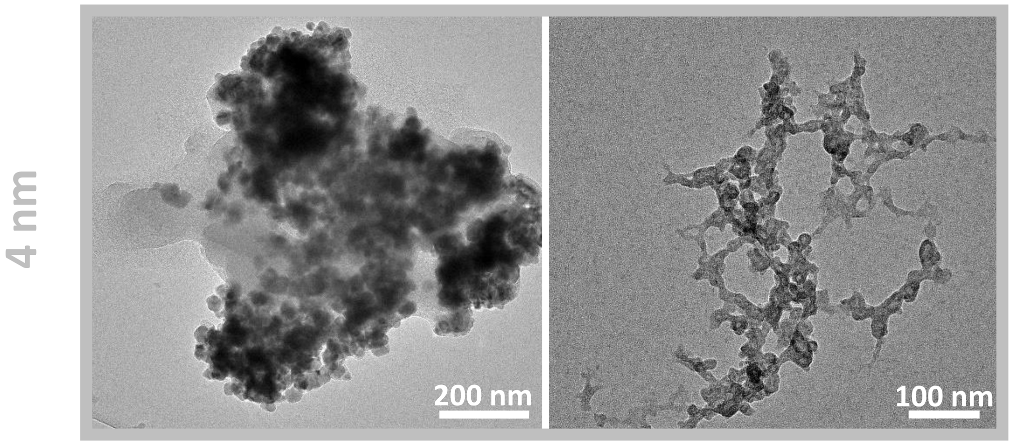

Figure S14. TEM images of used CuO 4 nm after 1h of CORR at -0.5V vs RHE.

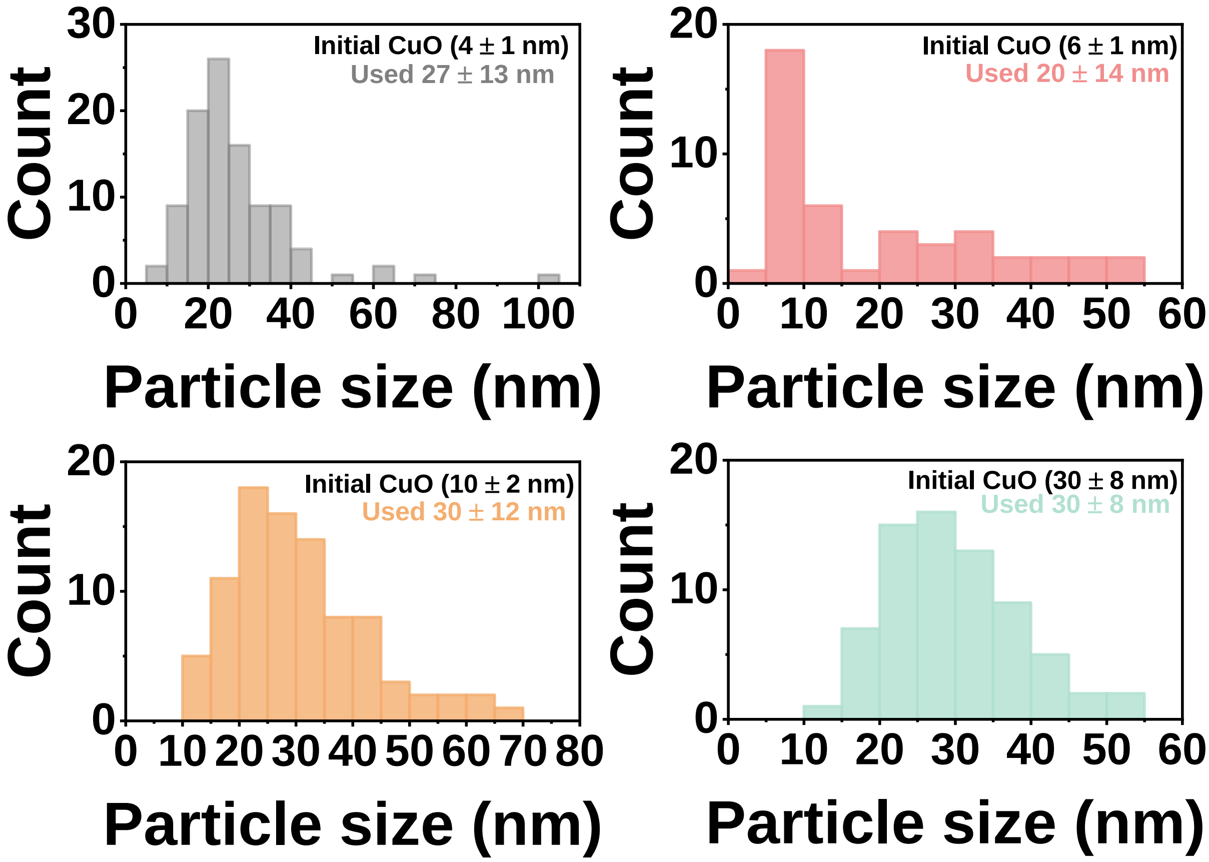

Figure S15. Particle size distributions of used samples (CuO initial size: 4nm, 6 nm, 10 nm and 30 nm) measured by TEM – Particles counted for 4 nm – 100 particles, 6 nm – 45 particles, 10 nm – 90 particles and 30 nm – 70 particles.

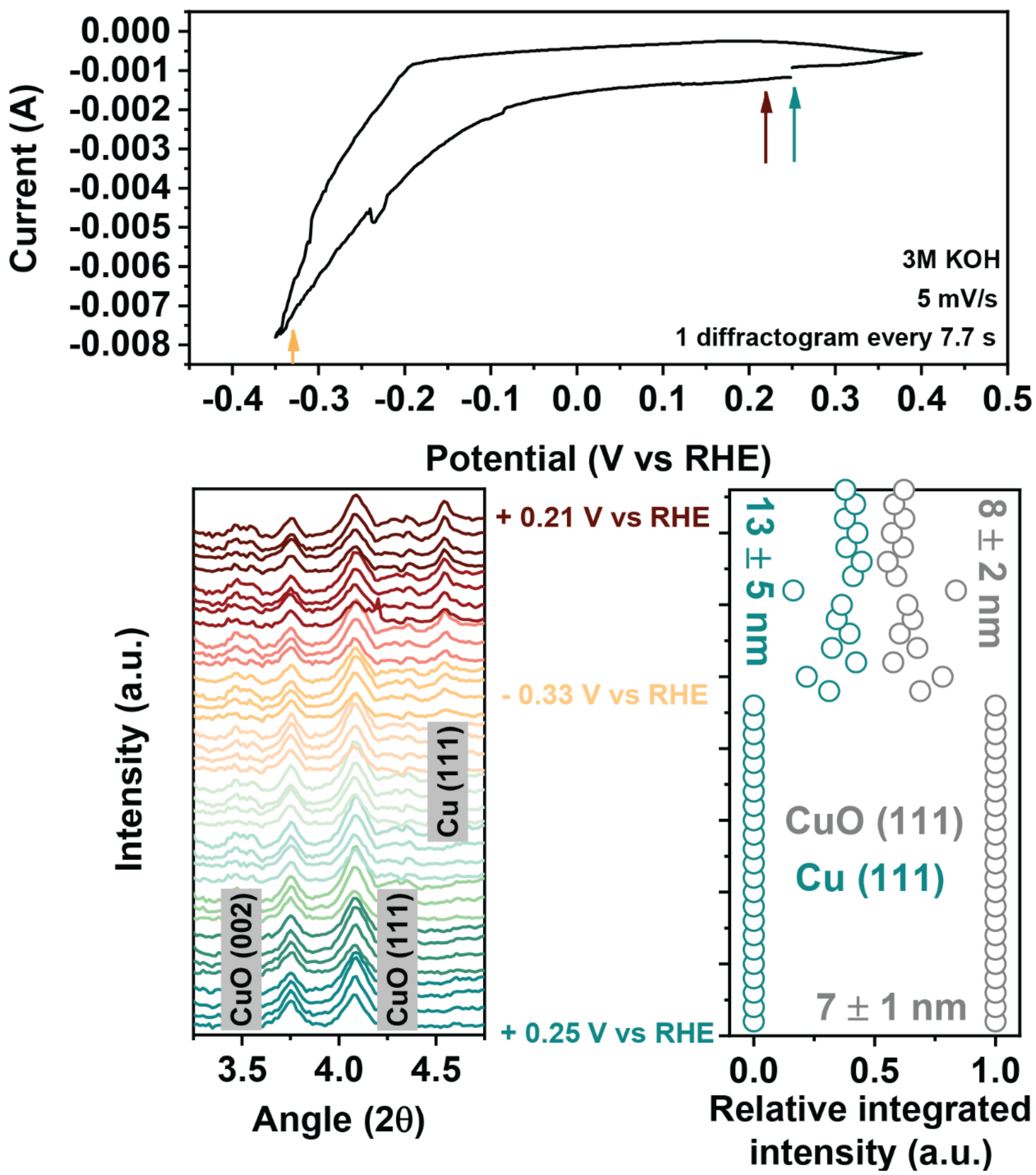

Figure S16. WAXS measurements and cyclic-voltammogram (1st cycle) recorded between +0.4 V vs RHE and -0.35 V vs RHE (4 nm).

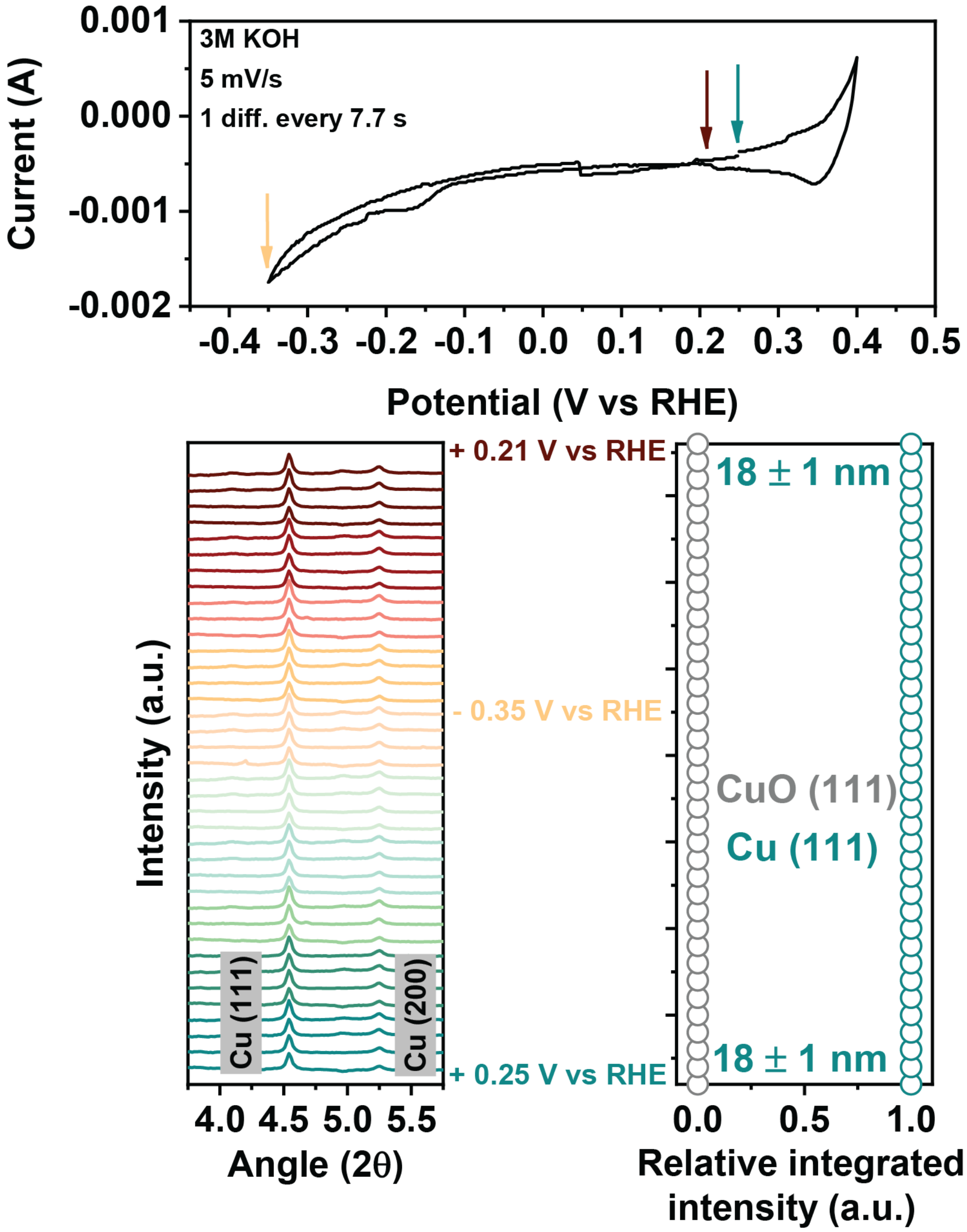

Figure S17. WAXS measurements and cyclic-voltammogram (last cycle) recorded between +0.4 V vs RHE and -0.35 V vs RHE (4 nm).

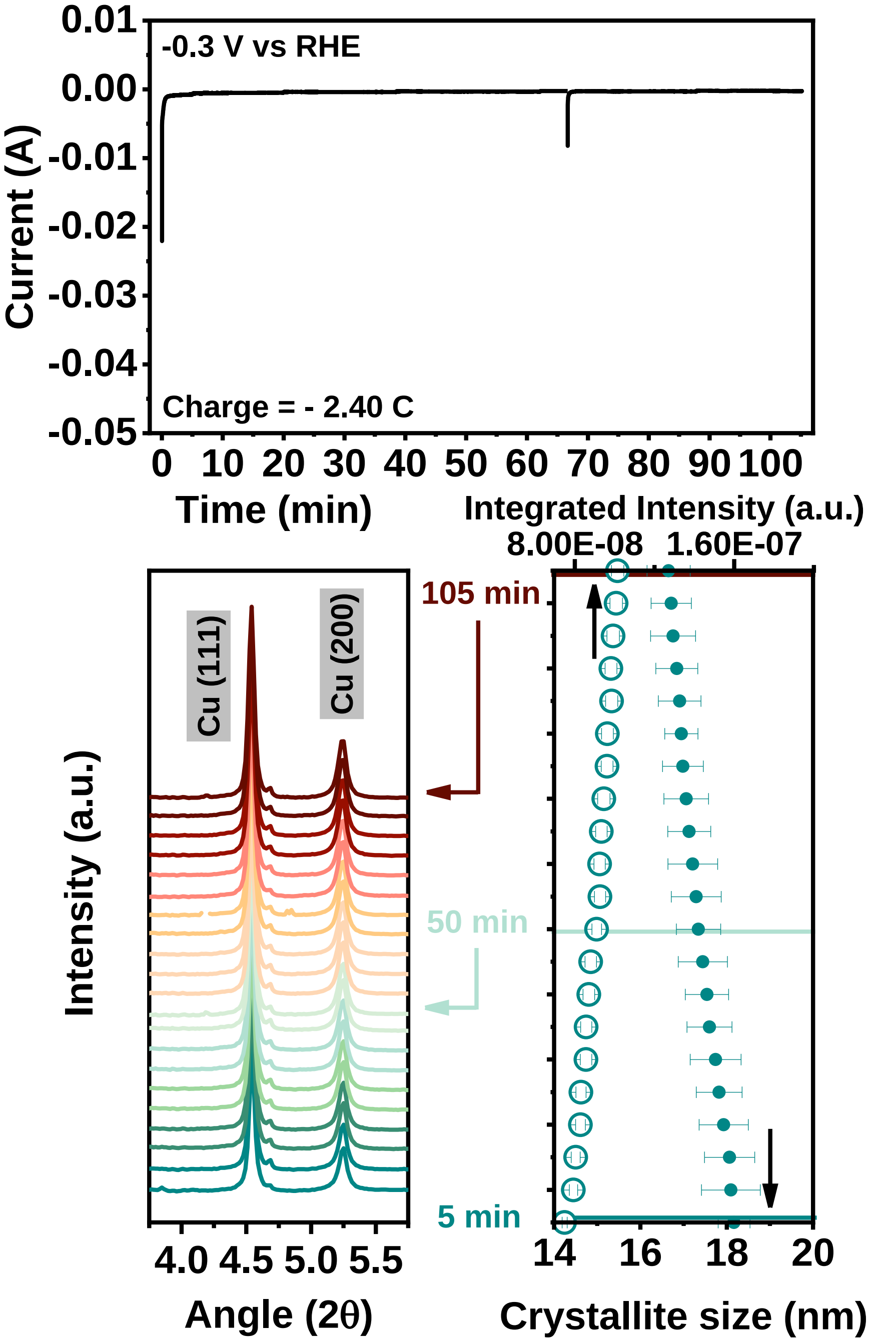

Figure S18. WAXS measurements and chronoamperometry of the 4 nm sample at -0.3 V vs RHE.

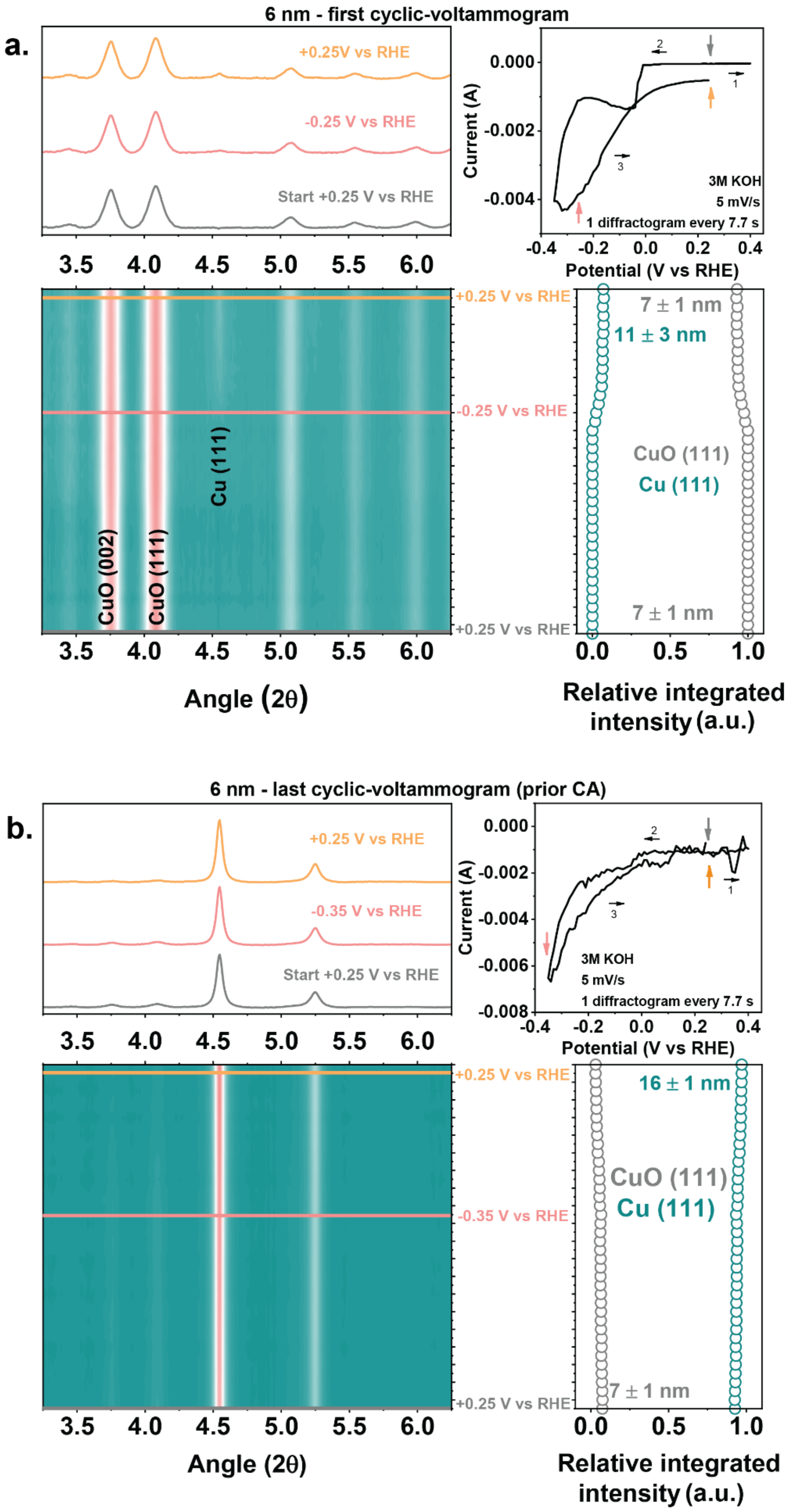

Figure S19. a) WAXS measurements and cyclic-voltammogram (1st cycle) recorded between +0.4 V vs RHE and -0.35 V vs RHE (6 nm), b) WAXS measurements and cyclic-voltammogram (last cycle) recorded between +0.4 V vs RHE and -0.35 V vs RHE (6 nm).

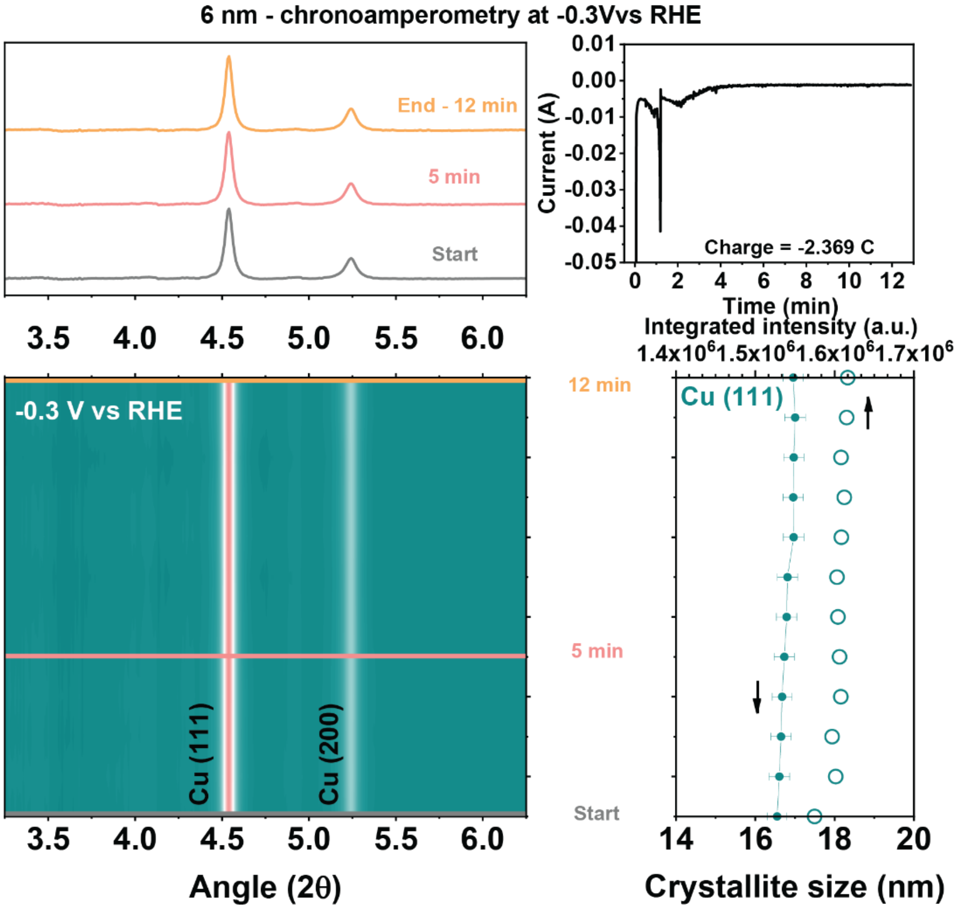

Figure S20. WAXS measurements and chronoamperometry of the 6 nm sample at -0.3 V vs RHE.

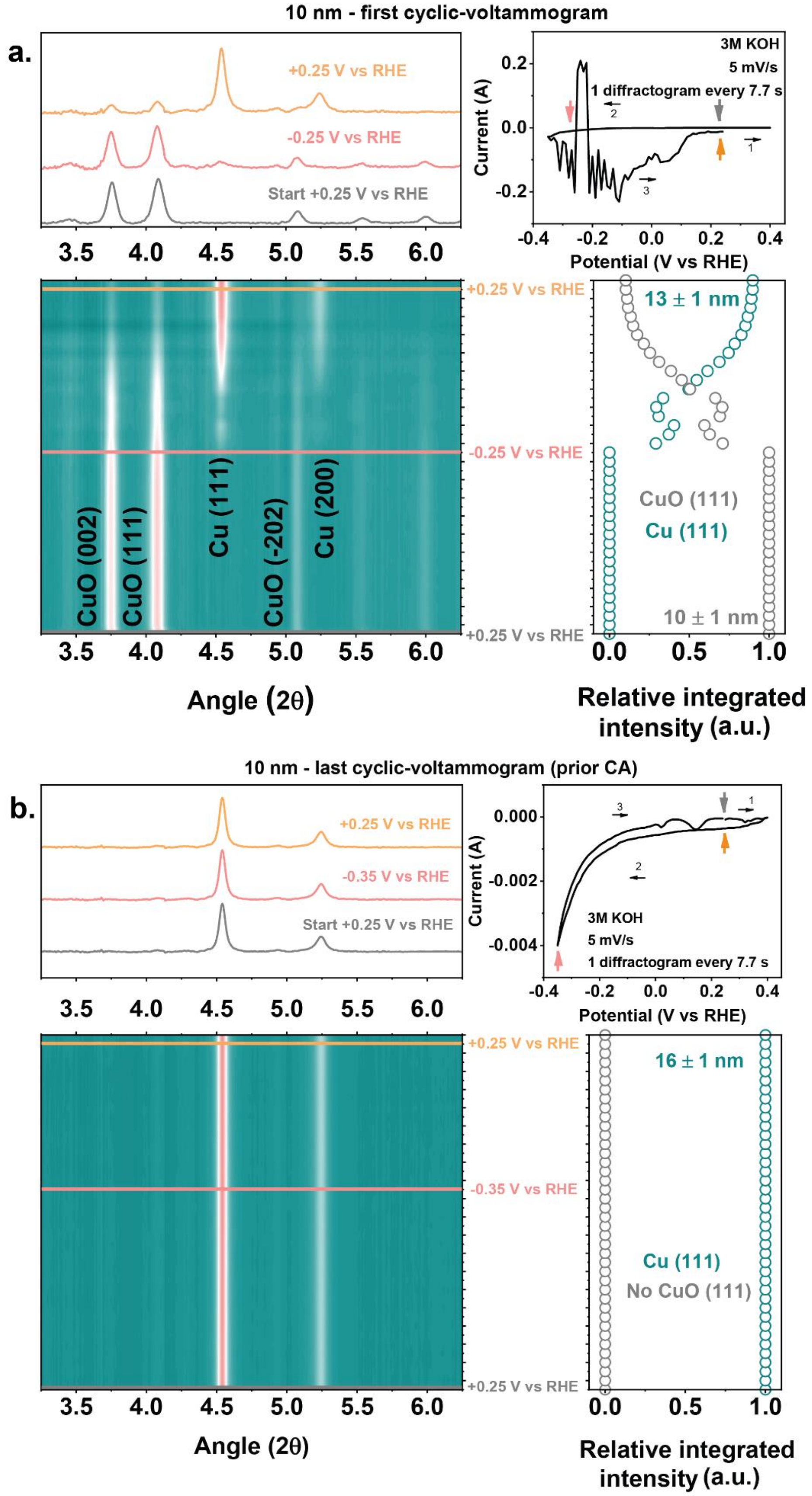

Figure S21. a) WAXS measurements and cyclic-voltammogram (1st cycle) recorded between +0.4 V vs RHE and -0.35 V vs RHE (10 nm), b) WAXS measurements and cyclic-voltammogram (last cycle) recorded between +0.4 V vs RHE and -0.35 V vs RHE (10 nm).

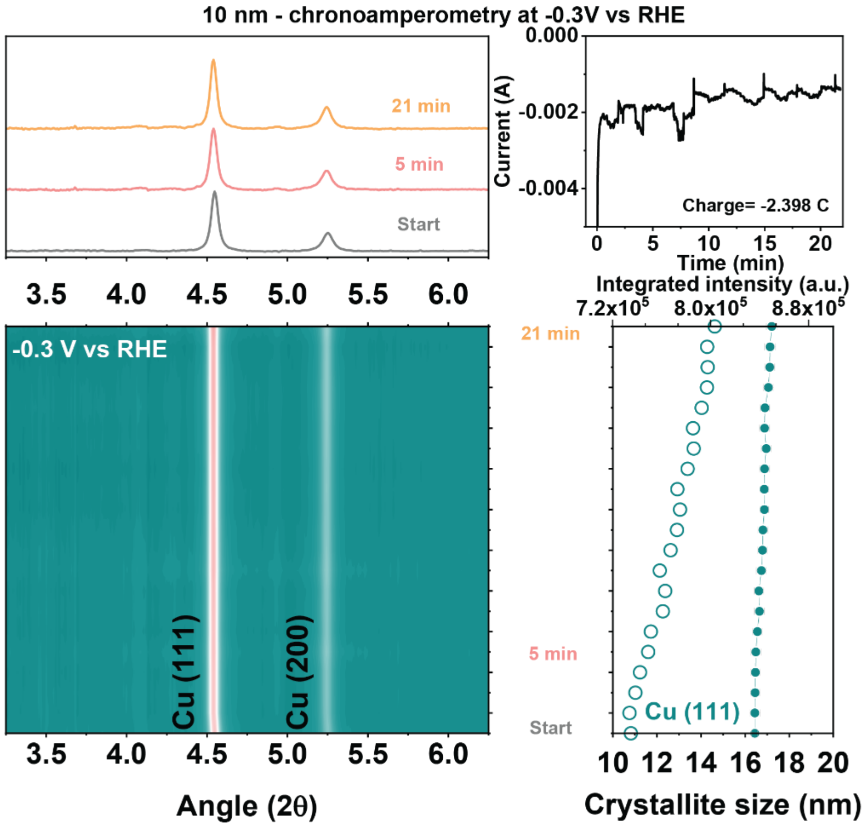

Figure S22. WAXS measurements and chronoamperometry of the 10 nm sample at -0.3 V vs RHE.

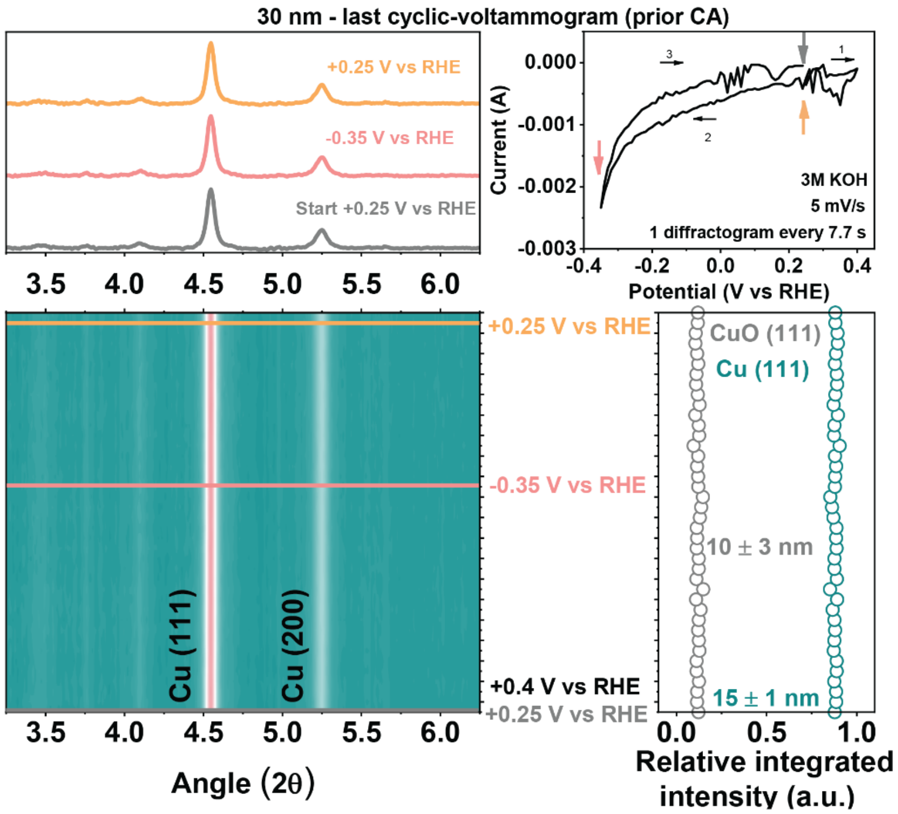

Figure S23. WAXS measurements and cyclic-voltammogram (last cycle) recorded between +0.4 V vs RHE and -0.35 V vs RHE (30 nm).

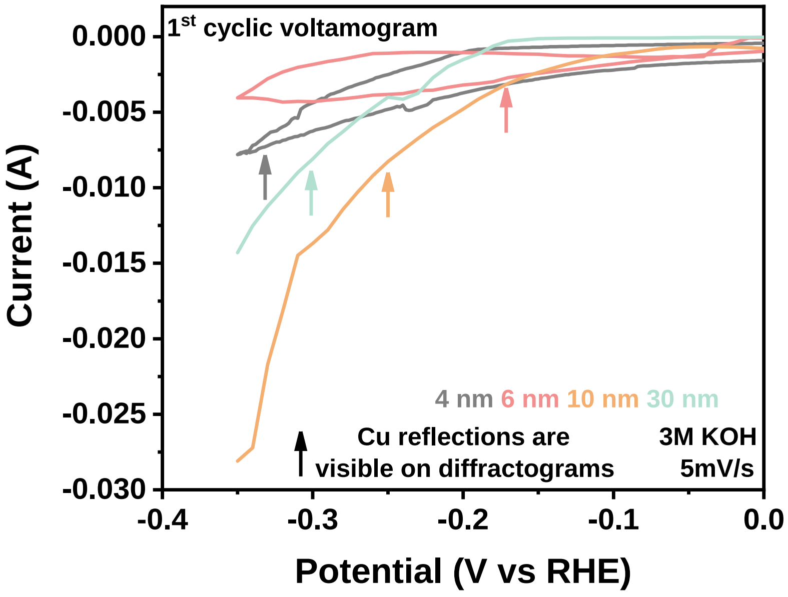

Figure S24. CV (1st cycle) of the samples recorded between +0.4 V and -0.35 V vs RHE during the WAXS experiments.

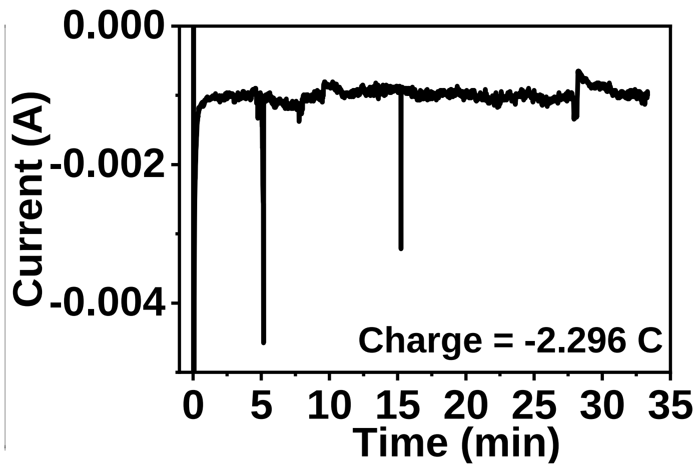

Figure S25. Chronoamperometry measurements of 30 nm sample at -0.3 vs RHE.

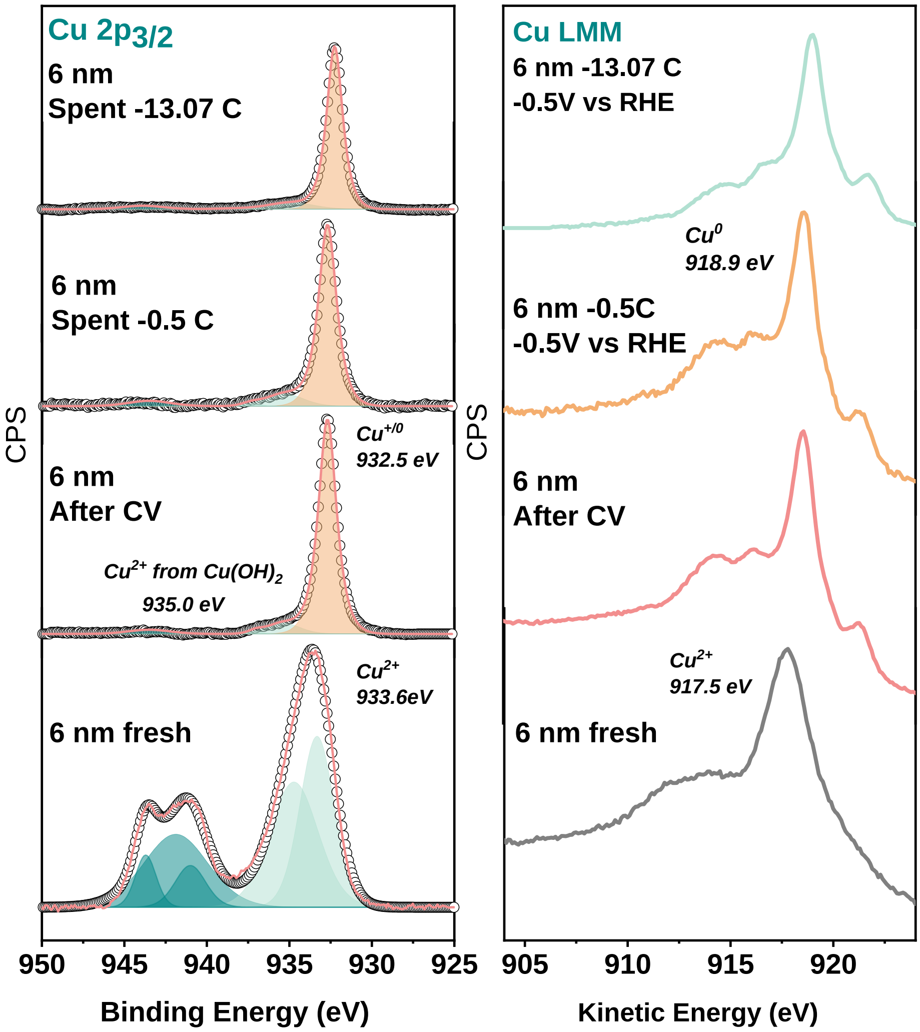

Figure S26. Quasi-in situ measurements of the 6 nm sample.

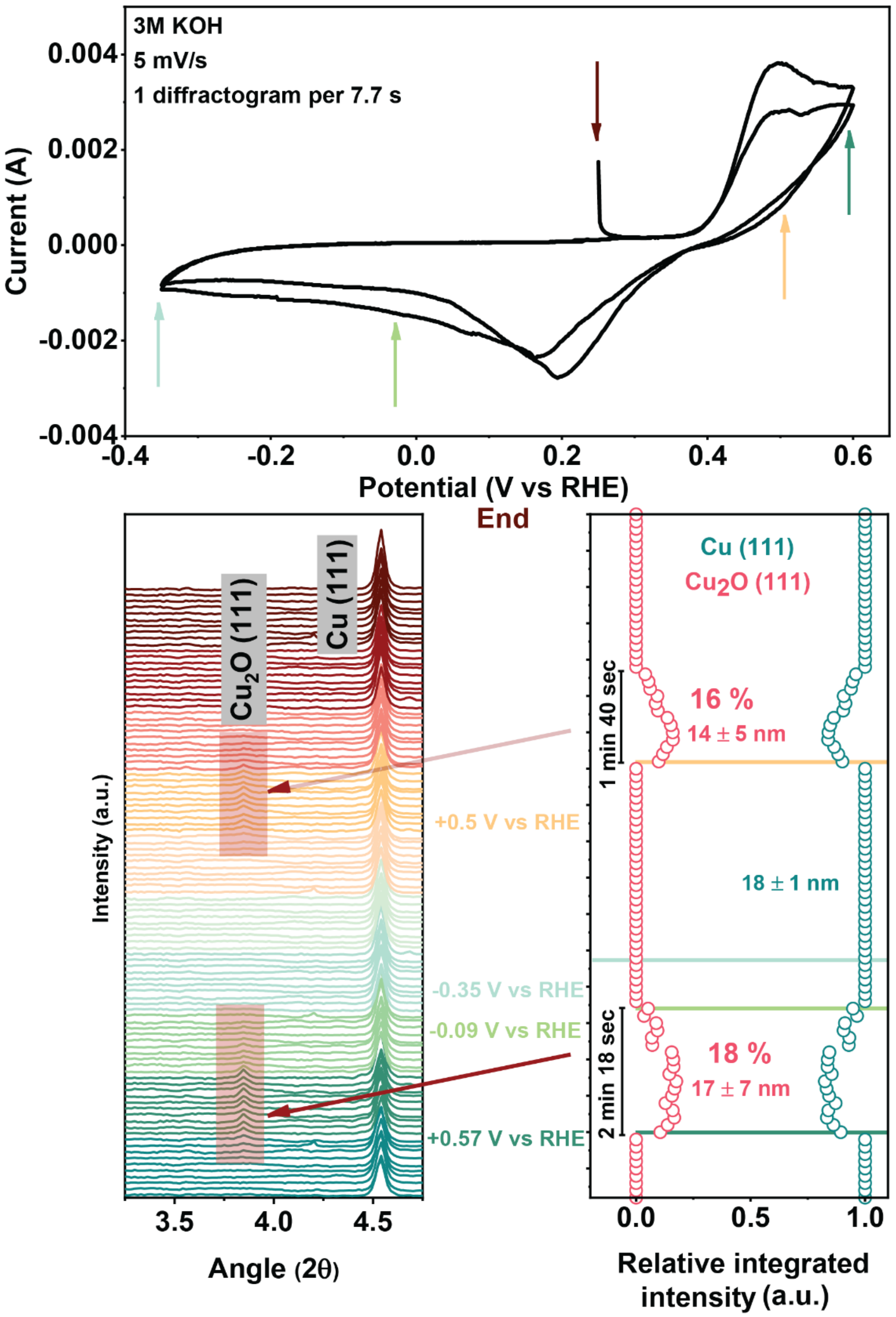

Figure S27. Electrochemical oxidation - WAXS and cyclic-voltammograms (2 cycles – 1st and 2nd CV cycle) ranging from +0.6 V vs RHE to -0.35 V vs RHE of the 4 nm sample.

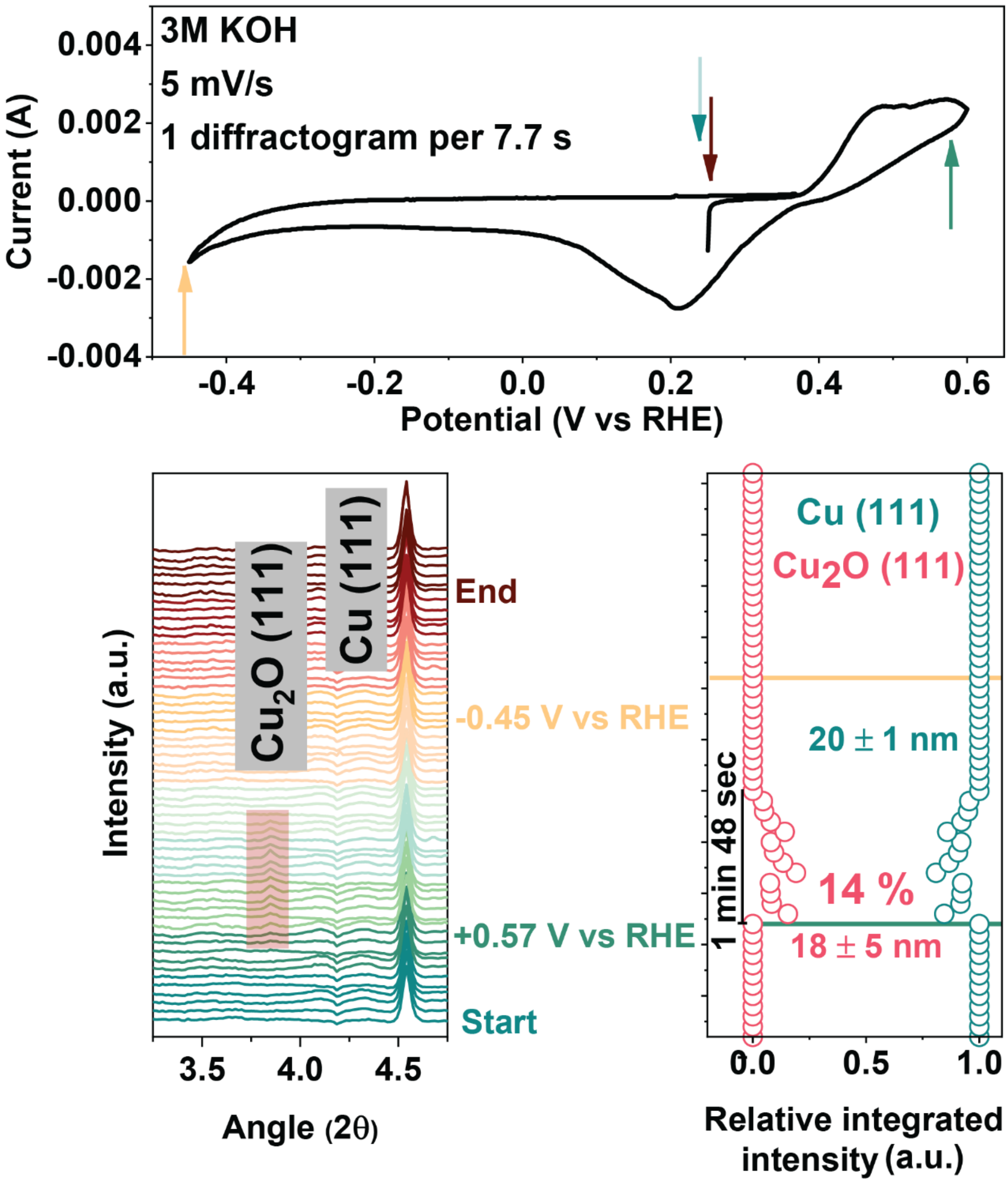

Figure S28. Electrochemical oxidation - WAXS and cyclic-voltammograms (1 cycle – 3rd CV cycle) ranging from +0.6 V vs RHE to -0.45 V vs RHE of the 4 nm sample.

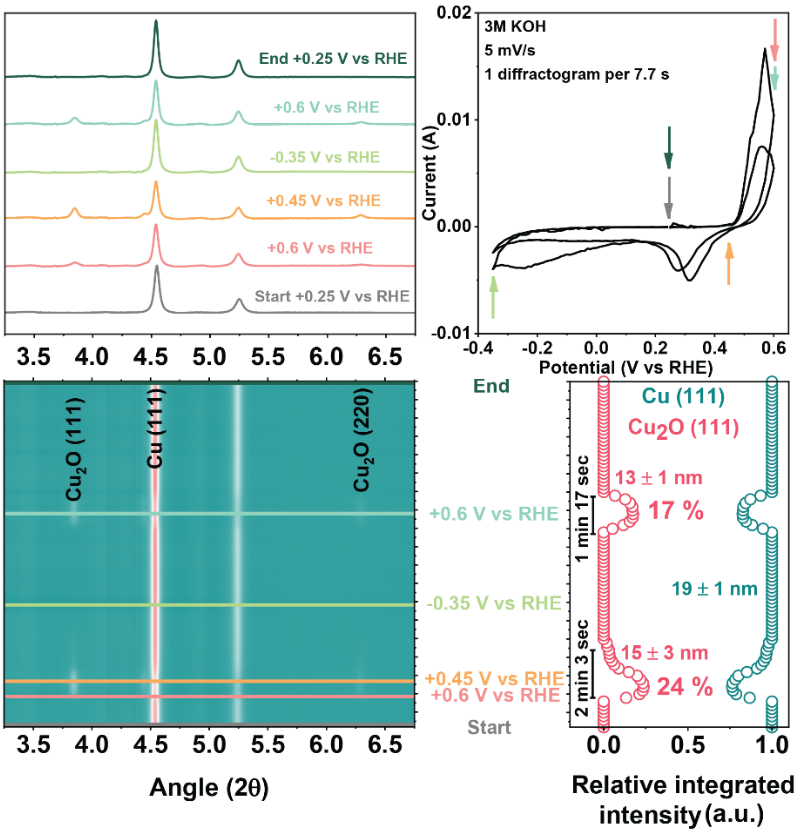

Figure S29. Electrochemical oxidation - WAXS and cyclic-voltammograms (2 cycles) ranging from +0.6 V vs RHE to -0.35 V vs RHE of the 6 nm sample.

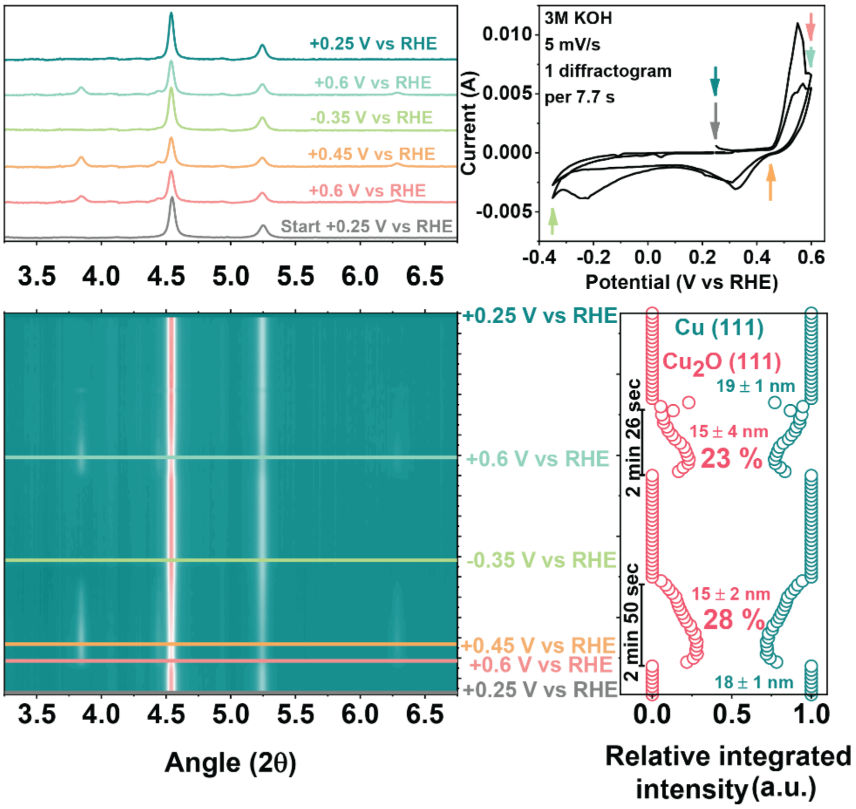

Figure S30. Electrochemical oxidation - WAXS and cyclic-voltammograms (2 cycles) ranging from +0.6 V vs RHE to -0.35 V vs RHE to study the redox properties of the 10 nm sample.

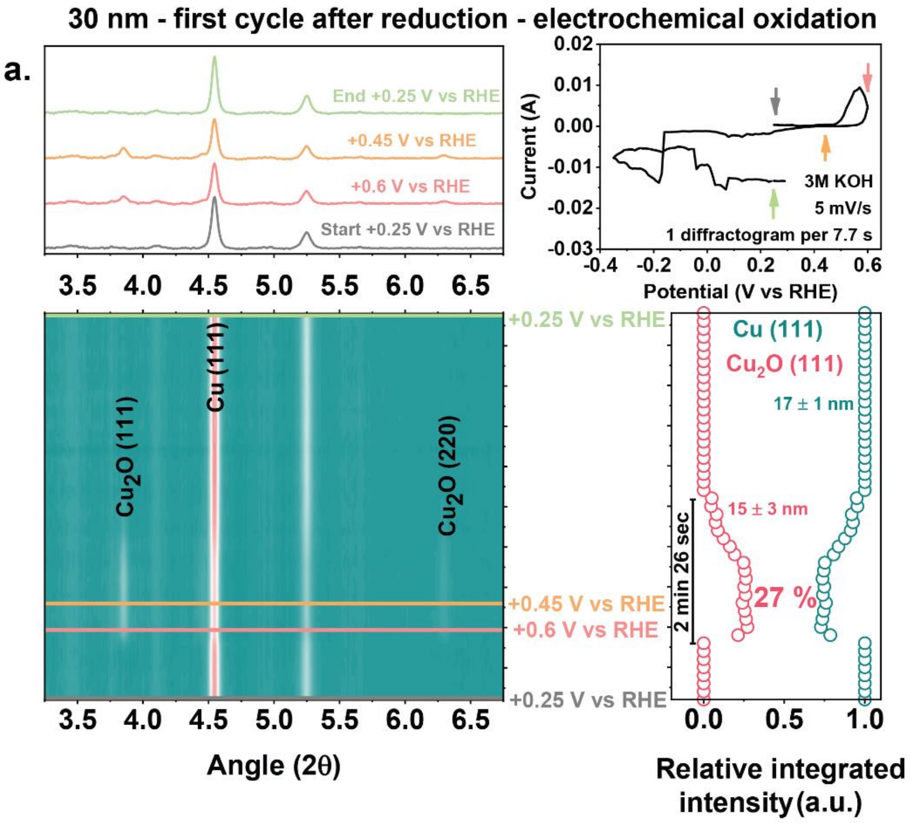

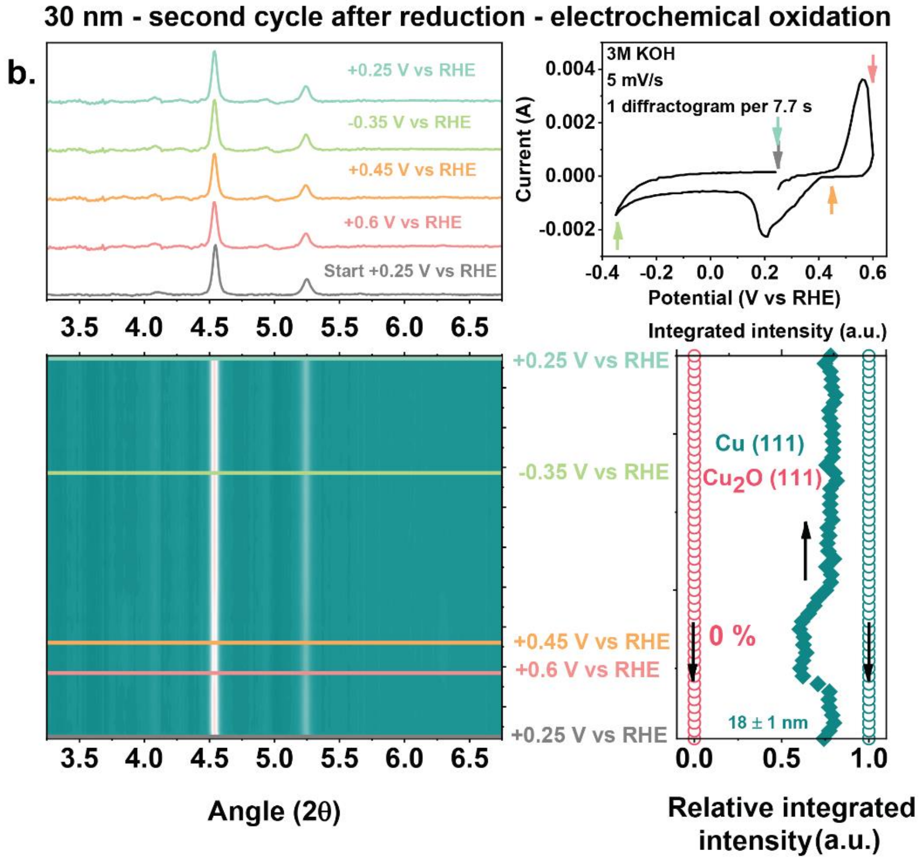

Figure S31. Electrochemical oxidation - a) WAXS and cyclic-voltammogram (1st cycle) ranging from +0.6 V vs RHE to -0.35 V vs RHE to study the redox properties of the 30 nm sample, b) WAXS and cyclic-voltammogram (2nd cycle) ranging from +0.6 V vs RHE to -0.35 V vs RHE to study the redox properties of the 30 nm sample.

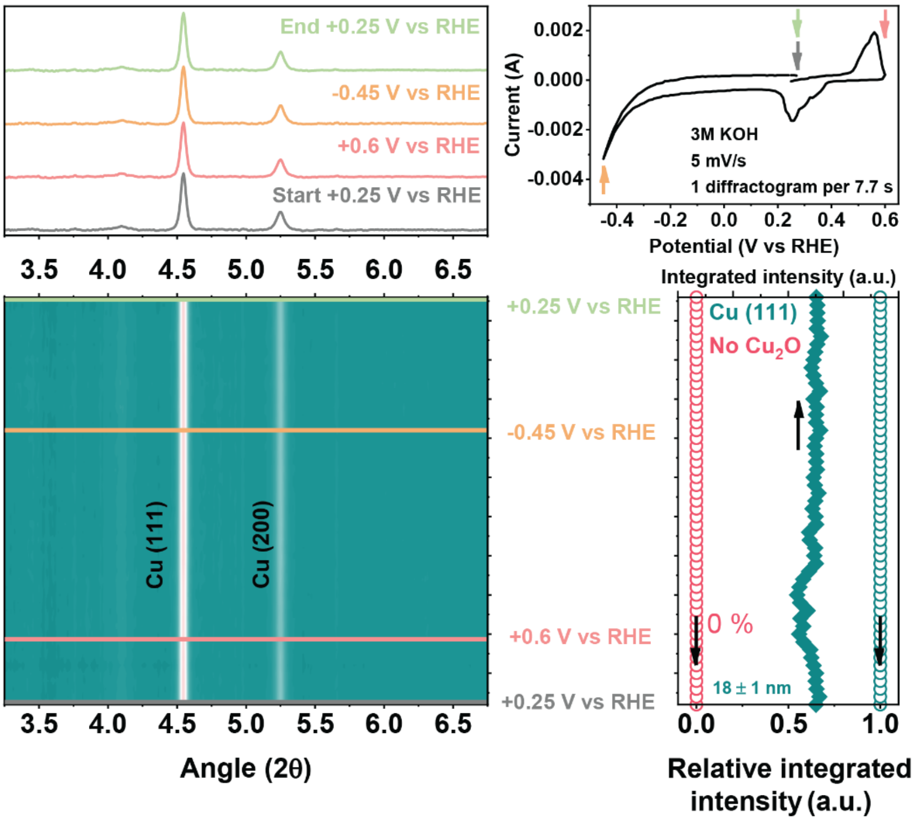

Figure S32. Electrochemical oxidation - WAXS and cyclic-voltammogram (last cycle) ranging from +0.6 V vs RHE to -0.45 V vs RHE to study the redox properties of the 30 nm sample.

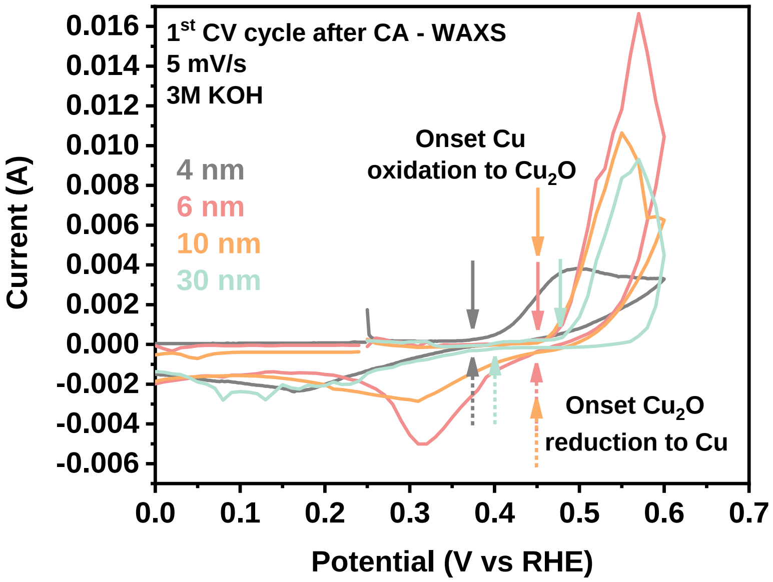

Figure S33. Electrochemical oxidation - Cyclic-voltammograms (first cycle after CA) ranging from +0.6 V vs RHE to 0.0 V vs RHE.

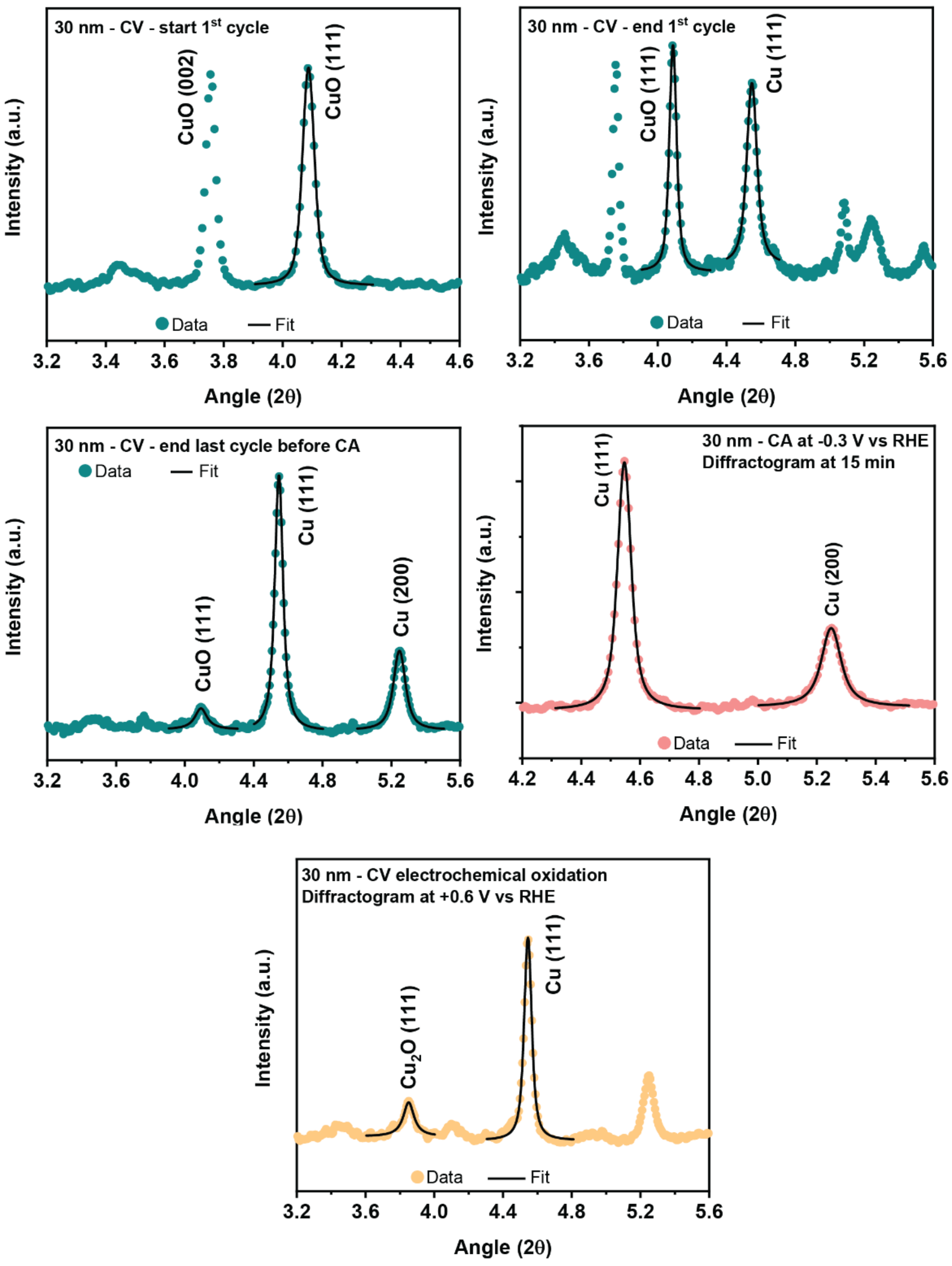

Figure S34. Examples of fitting of the reflections of the 30 nm sample.

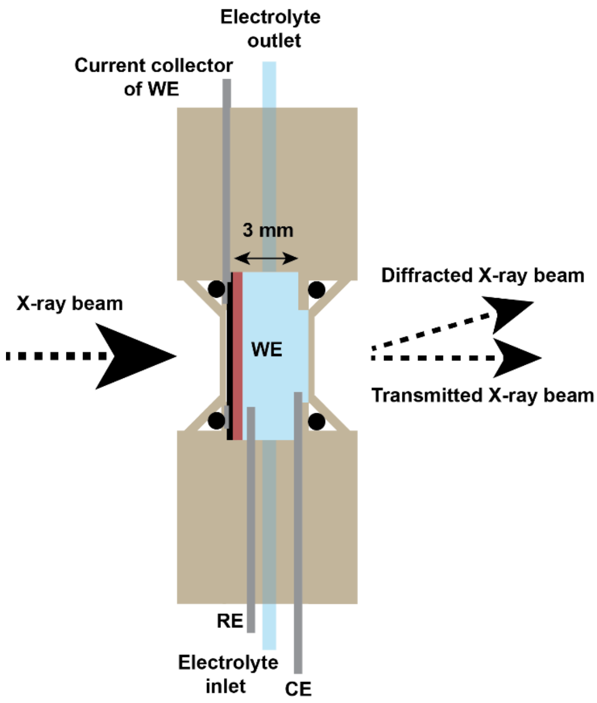

Figure S35. Schematics of the homemade WAXS cell.

References

(1) Biesinger, M. C. Advanced Analysis of Copper X-Ray Photoelectron Spectra. *Surface and Interface Analysis* 2017, *49* (13), 1325–1334. https://doi.org/10.1002/SIA.6239.

(2) McCrory, C. C. L.; Jung, S.; Peters, J. C.; Jaramillo, T. F. Benchmarking Heterogeneous Electrocatalysts for the Oxygen Evolution Reaction. *J Am Chem Soc* 2013, *135* (45), 16977–16987. https://doi.org/10.1021/JA407115P.

(3) Hegde, C.; Sun, X.; Dinh, K. N.; Huang, A.; Ren, H.; Li, B.; Dangol, R.; Liu, C.; Wang, Z.; Yan, Q.; Li, H. Cu- And Fe-Codoped Ni Porous Networks as an Active Electrocatalyst for Hydrogen Evolution in Alkaline Medium. *ACS Appl Mater Interfaces* 2020, *12* (2), 2380–2389. https://doi.org/10.1021/acsami.9b17273.

(4) Speck, F. D.; Cherevko, S. Electrochemical Copper Dissolution: A Benchmark for Stable CO_2 Reduction on Copper Electrocatalysts. *Electrochem commun* 2020, *115*, 106739. https://doi.org/10.1016/J.ELECOM.2020.106739.

(5) Vavra, J.; Dattila, F.; Kormányos, A.; Cherevko, S.; Lopéz, N.; Buonsanti, R. Cu^{+} Transient Species Mediate Cu Catalyst Reconstruction during CO_2 Electroreduction. ChemRxiv repository 2023. https://doi.org/10.26434/CHEMRXIV-2022-3CR9K.

(6) Tiwari, A.; Heenen, H. H.; Bjørnlund, A. S.; Hochfilzer, D.; Chan, K.; Horch, S. Electrochemical Oxidation of CO on Cu Single Crystals under Alkaline Conditions. *ACS Energy Lett* 2020, *5* (11), 3437–3442. https://doi.org/10.1021/ACSENERGYLETT.0C01751.

(7) Vavra, J.; Shen, T. H.; Stoian, D.; Tileli, V.; Buonsanti, R. Real-Time Monitoring Reveals Dissolution/Redeposition Mechanism in Copper Nanocatalysts during the Initial Stages of the CO2 Reduction Reaction. *Angewandte Chemie International Edition* 2021, *60* (3), 1347–1354. https://doi.org/10.1002/ANIE.202011137.

(8) Ma, M.; Kim, S.; Chorkendorff, I.; Seger, B. Role of Ion-Selective Membranes in the Carbon Balance for CO 2 Electroreduction via Gas Diffusion Electrode Reactor Designs. *Chem Sci* 2020, *11* (33), 8854–8861. https://doi.org/10.1039/D0SC03047C.

(9) Nitopi, S.; Bertheussen, E.; Scott, S. B.; Liu, X.; Engstfeld, A. K.; Horch, S.; Seger, B.; Stephens, I. E. L.; Chan, K.; Hahn, C.; Nørskov, J. K.; Jaramillo, T. F.; Chorkendorff, I. Progress and Perspectives of Electrochemical CO2 Reduction on Copper in Aqueous Electrolyte. *Chemical Reviews*. 2019, 119, 12, 7610-7672. https://doi.org/10.1021/acs.chemrev.8b00705.
